# High-throughput sequencing analysis of microbial community diversity in response to *indica* and *japonica bar*-transgenic rice paddy soils

**DOI:** 10.1371/journal.pone.0222191

**Published:** 2019-09-09

**Authors:** Meidan He, Jiachao Zhang, Linbo Shen, Lixin Xu, Wenjie Luo, Dong Li, Nanxin Zhai, Jianfa Zhao, Yan Long, Xinwu Pei, Qianhua Yuan

**Affiliations:** 1 Hainan Key Laboratory for Sustainable Utilization of Tropical Bio-resources, Institute of Tropical Agriculture and Forestry, Hainan University, Haikou, China; 2 College of Food Science and Technology, Hainan University, Haikou, China; 3 Institute of Tropical Bioscience and Biotechnology, Chinese Academy of Tropical Agriculture, Haikou, China; 4 Ministry of Agriculture Key Laboratory on Safety Assessment (Molecular) of Agriculture Genetically Modified Organisms, Biotechnology Research Institute, Chinese Academy of Agriculture Sciences, Beijing, China; Huazhong University of Science and Technology, CHINA

## Abstract

Potential environmental risks of genetically modified (GM) crops have raised concerns. To better understand the effect of transgenic rice on the bacterial community in paddy soil, a field experiment was carried out using pairs of rice varieties from two subspecies (*indica* and *japonica*) containing *bar* transgene with herbicide resistance and their parental conventional rice. The 16S rRNA gene of soil genomic DNA from different soil layers at the maturity stage was sequenced using high-throughput sequencing on the Illumina MiSeq platform to explore the microbial community diversity among different rice soils. There were no significant differences in diversity indices between transgenic *japonica* rice and its sister conventional rice (*japonica* pair) among different soil layers, but, significant differences was observed between transgenic *indica* rice and its conventional rice (*indica* pair) in the topsoil layer around concentrated rice roots according to the ace diversity index. Though the *japonica* rice soil and *indica* rice soil were shared several key genera, including *Rivibacter*, *Anaeromyxobacter*, *Roseomonas*, *Geobacter*, *Thiobacillus*, *Clostridium*, and *Desulfobulbus*, the primary bacterial genera in *indica* rice soil were different from those in *japonica* rice. *Synechococcus* and *Dechloromonas* were present in *japonica* rice samples, while *Chloronema*, *Flexibacter*, and *Blastocatella* were observed in *indica* rice soil. Moreover, the abundance of genera between GM and non-GM varieties in *japonica* rice was significantly different from *indica* rice, and several bacterial communities influenced these differences. *Anaerovorax* was more abundant in transgenic *japonica* rice soil than conventional rice soil, while it was deficient in transgenic *indica* rice soil compared to conventional rice soil, and opposite responses to *Deferrisoma* were in that of *indica* rice. Thus, we concluded that transgenic *indica* and *japonica* rice had different effects on soil bacteria compared with their corresponding sister conventional rice. However, these composition and abundance difference only occurred for a few genera but had no effect on the primary genera and soil characteristics were mainly contributed to these differences. Thus, differences in bacterial community structure can be ignored when evaluating the impacts of transgenic rice in the complex soil microenvironment.

## Introduction

Since genetically modified organisms (GMOs) developed and GM crops commercialized in 1996, the global area where biotech crops are planted has sharply increased. Up to 17 million farmers in 24 countries planted 189.8 million hectares of biotech crops in 2017 [1]. GM crops are considered to be the most rapidly adopted crop in modern times [2]. Nevertheless, scientists were worried about the potential risks of GMOs due to diversification of exogenous gene sources and the unpredictability of biological development [3–6]. The potential risks of GMOs have also received scrutiny from consumers, making assessments of GMOs contained in food and feed more important [5–8].

Soil microbial community is an important ecological indicator when accessing the safety of transgenic crops [9]. Due to the interposition of an exogenous gene, GM crops may affect the soil microbial community through their exudates and residues. Numerous, recent studies on interactions between microorganisms and plants had found that microbial diversity of the soil ecosystem is primarily confined to the rhizosphere. To determine the main effects on soil microorganisms from GM crops, researchers had used multiple approaches to conduct comprehensive analyses of transgenic potato producing T4 lysozyme for protection against bacterial infections. Heuer et al. found that environmental factors were the primary influence on the rhizosphere communities, but the effects of T4 lysozyme released from transgenic potato roots did not affect these communities [10]. *Bacillus thuringiensis* (*Bt*) crops with insect resistance did not have disadvantageous effects on the composition or activity of the microbial community when planted in field conditions [11–14]. With the ribosomal 16S rRNA gene pyrosequencing, a GM maize, containing three insecticidal proteins in the root tissue, which did not affect the rhizosphere bacterial community compared to conventional varieties [15]. Drought-tolerant *CaMSRB2*-expressing transgenic rice had a similar contribution to soil microorganisms as the parental rice based on pyrosequencing analysis [16]. Salt-tolerant *SUV3* overexpressing transgenic rice was healthy and had equivalent functionality (i.e. the activity of bacterial enzymes and plant growth promotion) to the rhizosphere microorganisms as non-transgenic rice [17]. Beta-carotene transgenic rice with four synthetic carotenoid genes had similar effects on bacterial communities and enzyme activities in the rhizosphere to its parent lines [18]. However, cultivation of drought-tolerant and insect-resistant rice had remarkable effect on bacterial community composition [19].

Rhizosphere bacterial diversity is influenced by both plant and soil type. Studies on rhizoplane microbial communities from genetically modified crops and control plants have shown that major differences occur among non-transgenic cultivars rather than between non-transgenic and transgenic lines [20]. However, Andreote et al. [21] observed an increase in *Erwinia* spp. and a decrease in *Agrobacterium* spp. associated with the transgenic plants. Zhu et al. [22] showed that the methanogenic archaeal community in the *Bt* rice rhizosphere was more active than that in the conventional rice varieties. The inconsistent results of the previous studies could be due to differences in materials, sites, and, approaches and technologies. Soil factors, plant root exudates and agricultural management are the major factors that determine community composition and abundance within the rhizosphere [23]. So far, the evaluation of impacts of GM crops on their soil microenvironments have mostly focused on insect resistance and other functional characteristics, while there have been few assessments related to herbicide resistance. The bialaphos resistance (*bar*) gene which was isolated from bacteria *Streptomyces hygroscopicus* confers tolerance to the herbicide glufosinate, has been globally used in basic plant research and genetically engineered crops [24, 25]. *Bar* gene is also considered to be a useful marker for the selection of transgenic plants [26]. Despite benefits like this, it is essential to investigate the potential environmental risk of transgenic rice before commercial release [27]. Most early studies of soil microorganisms were based on a plate colony counting technique, physiological approaches, and uncultivable molecular assays (including PCR-DGGE, T-RFLP, and pyrosequencing) [16, 18, 28, 29]. Few studies have taken advantage of high-throughput sequencing technology on the Illumina MiSeq platform.

Rice (*Oryza sativa L*.) has a strategic role as a food source for humans. Rice paddies are important ecosystems. Environmental assessments of GM rice are not only helpful for commercialization of transgenic rice, but also for determining safety of subsequent crops, especially root and tuber crops. Cultivated rice is classified into *indica* rice (*O*. *sativa* L. subsp. *indica* Kato) and *japonica* rice (*O*. *sativa* L. subsp. *japonica* Kato), but till now, comparisons of the two subspecies or their GM/non-GM plants effect on soil microorganisms have not been conducted. In addition, rice roots are pooled on the surface during the late growth stage, which differs from the other growth stages. As yet, little research was carried out on soil microorganism community in different soil layers according to the root distribution.

In this study, we focused on soil microbes at the maturity stage. We carried out high-throughput sequencing of 16S rRNA on the Illumina MiSeq platform, to determine differences in the structure and population of rhizosphere soil microbial communities in transgenic and conventional rice paddy soil. We were also interested in identifying any differences in the effects of transgenic *indica* rice and *japonica* rice on soil bacteria with their parental conventional rice. Results of this study will provide a theoretical basis for evaluating the impact of genetically modified rice on the soil environment.

## Materials and methods

### Experimental design and sampling

The experiment was carried out in a paddy field at Hainan University in Haikou, China (20° 03' 27.16" N 110° 19' 5.26" E). Two pairs of stably inherited rice varieties were used in this study: herbicide resistant *bar*-transgenic *japonica* rice B2 and its conventional sister line Xiushui63 without herbicide resistance; and *bar*-transgenic *indica* rice B68-1 with herbicide resistance and its conventional *indica* sister line D68 without herbicide resistance. *Japonica* rice B2 containing *bar* gene was a sibling of *japonica* line L201 which was produced by *japonica* cv. Xiushui63 crossed with a homozygous line that with *bar* gene transferred into *japonica* rice cv. JingYin119 [30, 31]. B68-1 was developed by transferring *bar* gene into *indica* rice variety D68 [32]. B2 and B68-1 were obtained from the China Rice Research Institute and the Institute of Subtropical Agriculture and Ecology, Chinese Academy of Sciences, respectively. The four rice varieties were arranged in a randomized block design with three replicates each for 4 m × 4 m plots. The plant spacing was 20 cm × 20 cm. According to the rice root distribution in the 20 cm of plowed soil, soils around the roots were divided into four parts to distinguish between the horizontal and vertical distances to the basal part of rice stem: the soil layer in topsoil where horizontal distance was 0–10 cm and vertical distance was 0–10 cm from rice stem, which was considered as topsoil of concentrated roots (TC); the soil layer in topsoil, where horizontal distance was 10–20 cm and vertical distance was 0–10 cm from rice stem, was considered as topsoil of dispersed roots (TD); the soil layer in subsoil where the horizontal distance was 0–10 cm and vertical distance was 10–20 cm from rice stem, was defined as subsoil of concentrated roots (SC); and the soil layer in subsoil where horizontal distance and vertical distance were 10–20 cm from rice stem, which was defined as subsoil of dispersed roots (SD) (S1 Fig, S1 Table). In addition, three replicates of blank soils with no plants were sampled randomly at every five positions in the same experimental field (Table 1). Rhizosphere soils in each position were collected using the S-shaped method with a geotome, excluding the bulk soil without roots, and then, collecting the soil that shaking off from roots in five random positions and mixed together as a representative composite soil sample (about 500 g fresh weight) at each plot. All samples were collected at the rice maturity stage in June 2015.

**Table 1:** Rice varieties and sampling information.

Rice type	Rice variety	Sample	Sampling depth (cm)	Sampling distance from basal part of rice stem (cm)
*Bar*-transgenic *indica* rice	B68-1	TITC	0–10	0–10
		TITD	0–10	10–20
		TISC	10–20	0–10
		TISD	10–20	10–20
Conventional *indica* rice	D68	CITC	0–10	0–10
		CITD	0–10	10–20
		CISC	10–20	0–10
		CISD	10–20	10–20
*Bar-*transgenic *japonica* rice	B2	TJTC	0–10	0–10
		TJTD	0–10	10–20
		TJSC	10–20	0–10
		TJSD	10–20	10–20
Conventional *japonica* rice	Xiushui63	CJTC	0–10	0–10
		CJTD	0–10	10–20
		CJSC	10–20	0–10
		CJSD	10–20	10–20
Without rice	Blank topsoil	BTS	0–10	/
Without rice	Blank subsoil	BSS	10–20	/

TI, CI, TJ, and CJ were abbreviated for rice lines *bar*-transgenic *indica* rice B68-1, conventional *indica* rice D68, *bar-*transgenic *japonica* rice B2 and conventional *japonica* rice Xiushui63, respectively. The soil layer in topsoil, where horizontal distance was 0–10 cm and vertical distance was 0–10 cm from rice stem, was considered as topsoil of concentrated roots (TC); the soil layer in topsoil, where horizontal distance was 10–20 cm and vertical distance was 0–10 cm from rice stem, was considered as topsoil of dispersed roots (TD); the soil layer in subsoil, where the horizontal distance was 0–10 cm and vertical distance was 10–20 cm from rice stem, was considered as subsoil of concentrated roots (SC); and the soil layer in subsoil, where horizontal distance and vertical distance were 10–20 cm from rice stem, was defined as subsoil of dispersed roots (SD). BTS, topsoil of blank soil without planting rice where the vertical distance was 0–10 cm from rice stem, BSS, subsoil of blank soil without planting rice vertical distance was 10–20 cm from rice stem.

Each soil sample was placed in individual germfree bags, and immediately stored in a freezer and samples were processed as soon as possible to make sure that the soil microorganisms were closer to the *in situ* environment. Each sample was split into two unequal parts. One part was about two thirds of each sample for detection of soil nutrients, the other part was about one third for DNA extraction. The former was kept at room temperature, and the latter was kept at -80°C after removing the rice roots.

### Physical and chemical properties of soil

There were 18 treated sample groups with a total of 54 independent soil samples. Soil pH was measured in 1:2.5 (W/V) suspensions of soil in distilled water using pH meter (with the electric potential method) and chromic acid oxygen titration was used to measure organic matter (OM). Total nitrogen (TN), ammonium nitrogen (AN), and nitrate nitrogen (NN) content of soil were respectively measured by the semi-micro Macro Kjeldahl method [33], the indophenol blue spectrophotometric method, and UV spectrophotometry after NaCl extraction (1:5 (W/V) suspensions of soil in 2.0 mol/L NaCl solution). Meanwhile, soil available phosphorus (AP) and total phosphorus content (TP) were measured using Bray I extraction—molybdenum antimony spectrophotometric and acid soluble molybdenum antimony colorimetric assay. Simultaneously, total potassium content (TK) of soil and soil available potassium (AK) were extracted using flame photometric determination.

### DNA extraction and polymerase chain reaction

A total of 54 paddy soil samples was centrifuged at 10,000 ×g for 30 secs, and the precipitate was collected as rhizosphere soil and used for DNA extraction; each sample was processed separately. Soil genomic DNA was extracted using the Power Soil DNA Isolation Kit (MoBio Laboratories Inc., USA) following the manufacturer’s instructions. The concentration and quality of the DNA were determined using a spectrophotometer (NanoVue Plus, USA) and the DNA quality and integrity was checked by electrophoresis on a 0.8% agarose golden view gel. The DNA extracted from each soil sample served as a template for amplification of bacterial 16S rRNA gene sequences. A set of primers (forward primer, 338F: 5’-ACTCCTACGGGAGGCAGCA-3’ and reverse primer, 806R: 5’-GGACTACHVGGGTWTCTAAT-3’) [34, 35] were used to amplify the V3-V4 region of the bacterial 16S rRNA gene. After amplification, the PCR product was detected by electrophoresis on a 2% agarose golden view gel.

### Illumina MiSeq sequencing of the V3-V4 region of bacterial 16S rRNA genes

The PCR products were extracted from 2% agarose gels and purified using the AxyPrep DNA Gel Extraction Kit (Axygen Biosciences, Union City, CA, USA) according to the manufacturer’s instructions. DNA was quantified by QuantiFluor^™^-ST (Promega, USA). Purified products from all samples were homogenized and pooled in equimolar concentrations to construct a PE library. Paired-end sequencing (2×300) with the TruSeq Universal Adapter (5’-AATGATACGGCGACCACCGAGATCTACACTCTTTCCCTACACGACGCTCTTCCGATCT-3’) and the TruSeq Adapter (5’GATCGGAAGAGCACACGTCTGAACTCCAGTCAC ATCACG ATCTCGTATGCCGTCTTCTGCTTG-3’ (the index was shown in boldface)) was conducted on an Illumina MiSeq platform (Majorbio, Shanghai). Sequencing data had been deposited to the NCBI Sequence Read Archive under accession number SRP188606 (https://www.ncbi.nlm.nih.gov/sra/?term=SRP188606).

### Processing and analyzing of sequencing data

After obtaining raw sequencing data, optimized data for bioinformatics analysis was obtained by removing the adapter, barcode, and chimera sequences and correcting the sequence direction. Then, operational taxonomic unit (OTU) sequence analysis was performed. The OTUs with 97% similarity cutoff were clustered using UPARSE (version 7.0) [36] in USEARCH. Taxonomy information based on OTU can be used to carry out statistical analysis of community structure at each level of classification. Representative OTU sequences were analyzed using a taxonomic database (silva 128/16S_bacteria). Rarefaction analysis was conducted in Mothur v.1.30.1 [37] to calculate alpha diversity, including sobs, chao1, ace, Shannon Wiener, and Simpson’s diversity indices. Beta diversity analysis and phylogenetic analysis were based on UniFrac [38]. Based on the above analysis, a series of in-depth statistical and visual analyses of community structure and phylogeny were carried out. Principal co-ordinates analysis (PCoA) were carried out using the community ecology package, the Vegan 2.0 package was used to generate a PCoA figure, and Venn diagrams were generated by Venn Diagram, while Kernel density estimation, redundancy analysis (RDA), and the heat-map figures were generated in Vegan 2.0 in R [39].

### Statistical analysis

Statistical analysis was done using IBM SPSS Statistics v21. Post hoc tests with the Student-Newman-Keuls method were used for one-way analysis of variance (ANOVA).

## Results

### Physical and chemical characteristics of different soil samples

Soil pH value ranged from 6.71 to 7.09 in this study (Table 2). In TC and SD soil layers, *bar*-*japonica* rice was similar to conventional rice, this was also the case for the *indica* pair. At the TD and SC soil layers, *bar*-*japonica* rice was also similar to conventional rice, however, the pH of *bar*-*indica* soil was significantly different from that of conventional rice. There were clear, significant differences between *japonica* rice and *indica* rice soils at each soil layers. Organic matter content ranged from 8.92 g/Kg to 17.98 g/Kg, and the OM, TP, and AP contents were not significantly different among all the compared groups. Total nitrogen at the TC soil layers only showed difference in that of the *indica* rice pair. The TK content of transgenic rice soils were almost the same as conventional samples. TK content of the *bar*-*indica* rice soil was significantly different from that of conventional rice at the TC soil layer, while the AK content showed some significant differences between the transgenic rice soils and their conventional pairs. For example, the AK content of *bar*-*indica* rice soil was significantly lower than that of conventional rice soil at the TC soil layer, while it was significantly higher than conventional samples at the SD soil layer. Interestingly, AN and NN content had a similar pattern: their content in *bar*-*japonica* rice soils was significantly lower than conventional rice soil at the TC and TD soil layers, while they were significantly higher than conventional rice soil at the SC and SD soil layers. Moreover, the AN content of *bar*-*indica* rice soil was significantly lower than in conventional rice soil at the SD soil layers.

**Table 2:** Physical and chemical characteristics of different soil samples.

Sample	pH	OM (g/kg)	TN (g/kg)	TP (g/kg)	TK (g/kg)	AP (mg/kg)	AK (mg/kg)	NN (mg/kg)	AN (mg/kg)
BST	7.03 ± 0.03 a	12.80 ± 1.2 ab	1.07 ± 0.11 a	0.74 ± 0.04 a	16.91 ± 1.12 a	21.2 ± 2.46 a	19.45 ± 2.5 c	3.83 ± 0.33 c	11.92 ± 1.3 c
TJTC	7.03 ± 0.04 a	14.30 ± 1.61 ab	0.91 ± 0.09 ab	0.75 ± 0.06 a	11.15 ± 1.26 bc	18.8 ± 1.29 a	47.35 ± 10.62 ab	41.63 ± 2.32 a	17.98 ± 1.64 b
CJTC	7.03 ± 0.01 a	16.18 ± 2.43 a	0.76 ± 0.09 bc	0.68 ± 0.06 a	10.14 ± 0.71 c	20.48 ± 8.19 a	36.71 ± 0.58 b	17.22 ± 2.16 b	26.49 ± 0.57 a
TITC	6.84 ± 0.04 b	11.50 ± 1.14 b	0.66 ± 0.10 c	0.59 ± 0.07 a	9.22 ± 1.26 c	15.17 ± 3.92 a	41.33 ± 3.53 b	4.81 ± 0.23 c	13.54 ± 1.35 bc
CITC	6.86 ± 0.04 b	12.51 ± 1.82 ab	0.98± 0.15 ab	0.75 ± 0.12 a	13.5 ± 0.7 b	25.36 ± 2.9 a	57.3 ± 10.37 a	3.62 ± 0.2 c	14.58 ± 3.78 bc
*p*-value	0.000	0.052	0.006	0.105	0.000	0.156	0.001	0.000	0.000
TJTD	7.06 ± 0.02 a	13.7 ± 0.32 a	0.91 ± 0.06 a	0.70 ± 0.07 a	10.72 ± 0.89 a	20.61 ± 2.5 a	63.17 ± 3.76 a	40.44 ± 1.32 a	28.58 ± 4.9 a
CJTD	7.06 ± 0.03 a	11.81 ± 0.32 ab	0.85 ± 0.04 a	0.68 ± 0.04 a	11.13 ± 1.22 a	20.76 ± 4.68 a	40.57 ± 3.58 b	15.11 ± 1.92 b	24.46 ± 5.01 b
TITD	6.76 ± 0.02 c	11.69 ± 1.57 ab	0.85 ± 0.1 a	0.70 ± 0.06 a	12.20 ± 2.27 a	19.56 ± 4.9 a	41.59 ± 5.8 b	4.44 ± 0.16 c	12.7 ± 0.13 c
CITD	6.85 ± 0.01 b	10.05 ± 0.84 b	0.71 ± 0.11 a	0.58 ± 0.1 a	13.23 ± 0.56 a	23.35 ± 5.67 a	36.08 ± 7.75 b	3.76 ± 0.23 c	15.27 ± 4.37 c
*p*-value	0.000	0.009	0.079	0.214	0.197	0.778	0.001	0.000	0.005
BSS	7.05 ± 0.03 a	11.1 ± 1.38 a	0.84 ± 0.11 a	0.76 ± 0.05 a	16.49 ± 0.27 a	43.38 ± 17.39 a	24.88 ± 11.18 c	3.68 ± 0.23 c	14.8 ± 3.37 c
TJSC	7.02 ± 0.04 a	12.88 ± 1.22 a	0.93 ± 0.1 a	0.69 ± 0.03 ab	11.20 ± 0.73 c	20.97 ± 3.02 b	69.81 ± 9.58 a	38.38 ± 4.03 a	25.48 ± 3.18 a
CJSC	7.05 ± 0.02 a	12.99 ± 4.34 a	0.74 ± 0.04 a	0.64 ± 0.02 b	11.05 ± 0.95 c	14.75 ± 2.87 b	47.68 ± 6.31 b	14.41 ± 2.53 b	20.42 ± 1.26 b
TISC	6.94 ± 0.01 b	11.18 ± 1.61 a	0.99 ± 0.21 a	0.7 ± 0.05 ab	13.18 ± 0.38 b	23.13 ± 3.28 b	46.25 ± 9.25 b	4.34 ± 0.22 c	13.29 ± 0.5 c
CISC	6.75 ± 0.03 c	10.09 ± 0.82 a	0.84 ± 0.05 a	0.64 ± 0.03 b	13.20 ± 0.94 b	21.14 ± 5.79 b	44.27 ± 1.78 b	3.85 ± 0.33 c	15.09 ± 1.9 c
*p*-value	0.000	0.489	0.196	0.018	0.000	0.019	0.001	0.000	0.000
TJSD	7.04 ± 0.04 a	11.39 ± 0.41 a	0.83 ± 0.09 a	0.67 ± 0.02 a	12.16 ± 1.42 a	16.70 ± 3.68 a	41.42 ± 6.31 b	34.07 ± 2.01 a	29.71 ± 3.95 a
CJSD	7.06 ± 0.03 a	11.34 ± 1.6 a	0.77 ± 0.09 a	0.71 ± 0.11 a	13.45 ± 1.79 a	26.81 ± 8.45 a	46.13 ± 2.79 b	16.37 ± 2.57 b	23.42 ± 1.03 b
TISD	6.77 ± 0.04 b	10.88 ± 0.37 a	0.90 ± 0.03 a	0.68 ± 0.03 a	13.48 ± 0.48 a	26.06 ± 1.07 a	71.79 ± 16.51 a	4.50 ± 0.22 c	12.08 ± 1.35 c
CISD	6.76 ± 0.06 b	9.60 ± 0.59 a	0.74 ± 0.1 a	0.57 ± 0.06 a	10.41 ± 3.80 a	19.58 ± 6.07 a	37.41 ± 7.03 b	3.58 ± 0.29 c	23.69 ± 2.60 b
*p*-value	0.000	0.124	0.171	0.135	0.352	0.145	0.009	0.000	0.000

AK, available potassium; AP, available phosphorus; AN, ammonium nitrogen; NN, nitrate nitrogen; OM, organic matter; TK, total potassium; TP, total phosphorus; TN, total nitrogen. TITC, topsoil of concentrated roots from *bar*-transgenic *indica* rice B68-1; TITD, topsoil of dispersed roots from *bar*-transgenic *indica* rice B68-1; TISC, subsoil of concentrated roots from *bar*-transgenic *indica* rice B68-1;TISD, subsoil of dispersed roots from *bar*-transgenic *indica* rice B68-1; CITC, topsoil of concentrated roots from conventional *indica* rice D68; CITD, topsoil of dispersed roots from conventional *indica* rice D68; CISC, subsoil of concentrated roots from conventional *indica* rice D68; CISD, subsoil of dispersed roots from conventional *indica* rice D68; TJTC, topsoil of concentrated roots from *bar-*transgenic *japonica* rice B2; TJTD, topsoil of dispersed roots from *Bar-*transgenic *japonica* rice B2; TJSC, subsoil of concentrated roots from *bar-*transgenic *japonica* rice B2; TJSD, subsoil of dispersed roots from *bar-*transgenic *japonica* rice B2; CJTC, topsoil of concentrated roots from conventional *japonica* rice Xiushui63; CJTD, topsoil of dispersed roots from conventional *japonica* rice Xiushui63; CJSC, subsoil of concentrated roots from conventional *japonica* rice Xiushui63; CJSD, subsoil of dispersed roots from conventional *japonica* rice Xiushui63; BTS, topsoil of blank soil without planting rice; BSS, subsoil of blank soil without planting rice. Data was shown by the average of samples (n = 3) and their standard deviation. Different letters following after the data indicated significant differences (*p*<0.05) based on Student-Newman-Keuls post hoc tests among compared sample groups.

### Taxonomy and alpha diversity analysis of sequenced data

A total of 2,062,254 raw sequences of 16S rRNA were generated from 54 soil samples after Illumina MiSeq sequencing. There were 1,630,398 high quality sequences following quality control (80% retention), and each sample had around 30,192 sequences for later analysis. The average sequence length was 437 bp (S2 Table). Based on the minimum sample sequence, the rhizosphere bacterial community contained 59 phyla, 141 classes, 266 orders, 497 families, 937 genera, 1969 species, and 5384 OTUs. The most abundant phyla in all samples were 14 unnamed phyla, Proteobacteria (30%), and Chloroflexi (20%).

Each sample contained an average of 2,719 OTUs, according to the Shannon curve analysis. With an increase in the number of sequenced bands, samples of microbial diversity plateaued and increasing the sequencing reads did not significantly increase the diversity of microorganisms (S2 Fig). Diversity indices (S3 Table) were typically not significantly different at the same soil layer, except at the TC soil layer there were significant differences of ace index between *bar*-transgenic *indica* rice and its parental conventional rice and shannon index between *bar*-transgenic *japonica* rice and its parental conventional rice (Table 3).

**Table 3:** Different diversity indices with subsampling by the minimum number of sample sequences.

Samples	Sobs	Ace	Chao	Shannon	Coverage	Simpson
BTS	2290.7 ± 460.2 a	3629.6 ± 150.8 a	3375.6 ± 471.9 a	6.55 ± 0.68 a	0.931 ± 0.009 a	0.007 ± 0.008 a
CITC	1902.0 ± 287.8 a	3004.3 ± 414.3 b	2867.5 ± 382.7 a	6.28 ± 0.24 a	0.943 ± 0.009 a	0.007 ± 0.001 a
TITC	2441.3 ± 270.2 a	3799.5 ± 155.7 a	3479.8 ± 263.7 a	6.74 ± 0.37 a	0.928 ± 0.004 a	0.005 ± 0.004 a
CJTC	2626.0 ± 117.0 a	3752.2 ± 240.6 a	3669.4 ± 156.7 a	7.04 ± 0.04* a	0.925 ± 0.005 a	0.002 ± 0.00 a
TJTC	2395.7 ± 113.8 a	3536.3 ± 90.4 a	3479.4 ± 86.0 a	6.67 ± 0.02 a	0.928 ± 0.002 a	0.004 ± 0.001 a
average	2331.1 ± 343.9	3544.4 ± 357.7	3374.4 ± 381.7	6.66 ± 0.40	0.931 ± 0.008	0.005 ± 0.004
*p*-value	0.089	0.014	0.077	0.222	0.053	0.577
CITD	2334 ± 243.4 a	3953.9 ± 121.5 a	3449.7 ± 244.1 a	6.57 ± 0.36 a	0.928 ± 0.004 a	0.006 ± 0.003 a
TITD	2238.3 ± 315.6 a	3559.7 ± 24.8 a	3295.8 ± 293.5 a	6.47 ± 0.43 a	0.932 ± 0.007 a	0.008 ± 0.006 a
CJTD	2265.0 ± 235.5 a	3309.2 ± 294.7 a	3225.1 ± 321.0 a	6.59 ± 0.25 a	0.933 ± 0.006 a	0.005 ± 0.002 a
TJTD	2125.3 ± 99.1 a	3341.2 ± 433.6 a	3113.5 ± 186.5 a	6.35 ± 0.04 a	0.936 ± 0.005 a	0.007 ± 0.000 a
average	2240.7 ± 216.6 a	3541.0 ± 353.4 a	3271.0 ± 260.3 a	6.50 ± 0.28 a	0.932 ± 0.006 a	0.006 ± 0.003 a
*p*-value	0.755	0.064	0.51	0.78	0.361	0.751
BSS	2461.7 ± 82.6 a	3494.8 ± 294 a	3434.0 ± 297.1 a	6.87 ± 0.12 a	0.931 ± 0.008 a	0.003 ± 0.001 a
CISC	2037.7 ± 326.2 a	3355.6 ± 120.7 a	3109.9 ± 426.5 a	5.83 ± 0.95 a	0.935 ± 0.007 a	0.049 ± 0.058 a
TISC	2486.3 ± 204.1 a	3560.4 ± 286.9 a	3521.7 ± 322.0 a	6.93 ± 0.19 a	0.929 ± 0.006 a	0.002 ± 0.001 a
CJSC	2372.3 ± 203.7 a	3412.2 ± 264.4 a	3387.6 ± 295.0 a	6.67 ± 0.47 a	0.932 ± 0.005 a	0.011 ± 0.014 a
TJSC	2345.7 ± 228.6 a	3290.0 ± 299.0 a	3316.2 ± 284.3 a	6.84 ± 0.29 a	0.935 ± 0.006 a	0.003 ± 0.002 a
average	2340.7 ± 251.2	3422.6 ± 242.7	3353.9 ± 313.2	6.63 ± 0.60	0.932 ± 0.006	0.014 ± 0.029
*p*-value	0.18	0.732	0.628	0.112	0.777	0.217
CISD	2558.0 ± 174.9 a	3685.7 ± 241.3 a	3595.6 ± 186.7 a	6.93 ± 0.20 a	0.926 ± 0.005 a	0.003 ± 0.001 a
TISD	2514.3 ± 203.1 a	3550.4 ± 217.4 a	3545.5 ± 172.0 a	6.92 ± 0.17 a	0.929 ± 0.004 a	0.003 ± 0.001 a
CJSD	2495.0 ± 181.7 a	3544.3 ± 168.4 a	3527.1 ± 207.1 a	6.81 ± 0.38 a	0.929 ± 0.003 a	0.006 ± 0.006 a
TJSD	2522.7 ± 33.7 a	3554.1 ± 52.7 a	3497.1 ± 75.7 a	6.97 ± 0.06 a	0.930 ± 0.002 a	0.002 ± 0.000 a
average	2522.5 ± 140.9	3583.6 ± 169.2	3541.3 ± 148.2	6.91 ± 0.21	0.928 ± 0.003	0.004 ± 0.003
*p*-value	0.97	0.752	0.906	0.846	0.633	0.557

TITC, topsoil of concentrated roots from *bar*-transgenic *indica* rice B68-1; TITD, topsoil of dispersed roots from *bar*-transgenic *indica* rice B68-1; TISC, subsoil of concentrated roots from *bar*-transgenic *indica* rice B68-1;TISD, subsoil of dispersed roots from *bar*-transgenic *indica* rice B68-1; CITC, topsoil of concentrated roots from conventional *indica* rice D68; CITD, topsoil of dispersed roots from conventional *indica* rice D68; CISC, subsoil of concentrated roots from conventional *indica* rice D68; CISD, subsoil of dispersed roots from conventional *indica* rice D68; TJTC, topsoil of concentrated roots from *bar-*transgenic *japonica* rice B2; TJTD, topsoil of dispersed roots from *Bar-*transgenic *japonica* rice B2; TJSC, subsoil of concentrated roots from *bar-*transgenic *japonica* rice B2; TJSD, subsoil of dispersed roots from *bar-*transgenic *japonica* rice B2; CJTC, topsoil of concentrated roots from conventional *japonica* rice Xiushui63; CJTD, topsoil of dispersed roots from conventional *japonica* rice Xiushui63; CJSC, subsoil of concentrated roots from conventional *japonica* rice Xiushui63; CJSD, subsoil of dispersed roots from conventional *japonica* rice Xiushui63; BTS, topsoil of blank soil without planting rice; BSS, subsoil of blank soil without planting rice. Data was shown by the average of samples (n = 3) and their standard deviation. Different letters following after the data indicated significant differences (*p* <0.05) based on Student-Newman-Keuls post hoc tests,* showed for differences of compared pairs by Dunnett T3 post hoc tests

### Soil microbe distribution between *indica* rice and *japonica* rice

Various bacteria were obtained at OTU taxonomic levels. Relative abundance of bacterial communities up to 0.5% was defined as a dominant genus. *Bacillus* was a primary bacterium in paddy soil. The dominant genus *Leptolyngbya*, *Roseomonas*, and *Spirochaeta* were observed among the blank soils, *indica* rice soils, and *japonica* rice soils. Besides, blank soil also contained dominant bacterial genera *Blastocatella*, *Bryobacter*, *Sphingomonas*, *Thiobacillus*, *Marmoricola*, *Phormidium*, *Roseiflexus*, *Lyngbya*, *Nitrospira*, and *Flexibacter*.

**Fig 1:**
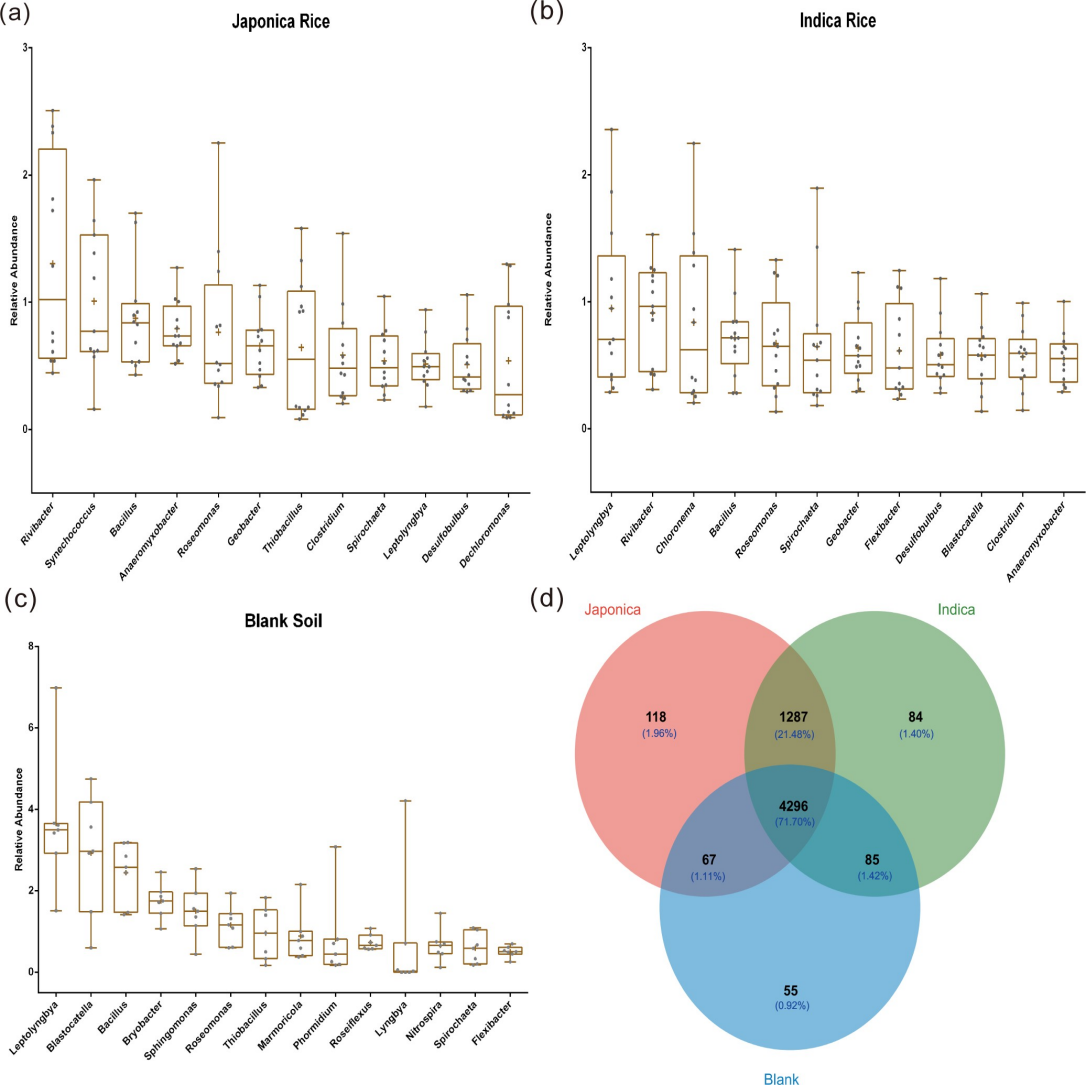
The primary bacterial genera, shared and unique OTU for three kinds of paddy soils. (a), the dominant bacterial genus in *japonica* rice soil. (b), the dominant bacterial genus in *indica* rice soil. (c), the dominant bacterial genus in blank rice soil. (d), the **s**hared and unique OTU for blank soil, *japonica* rice soil, and *indica* rice soil.

Though the *japonica* rice soil and *indica* rice soil were shared several key genera, including *Rivibacter*, *Anaeromyxobacter*, *Roseomonas*, *Geobacter*, *Thiobacillus*, *Clostridium*, and *Desulfobulbus*, however, dominant genera, *Synechococcus* and *Dechloromonas* were present in *japonica* rice samples, while *Chloronema*, *Flexibacter*, and *Blastocatella* were observed in *indica* rice soil. Dominant bacterial genera were shown in (Fig 1A, 1B and 1C).

Further analysis based on common microbial taxa showed that three kinds of paddy soil samples contained 4,296 OTUs, up to 71.70% of all OTUs. These shared OTUs mainly belonged to *Bacillus*, *Clostridium*, *Sphingomonas*, *Flexibacter*, *Blastocatella*, *Thiobacillus*, *Marmoricola*, *Leptolyngbya*, and *Anaeromyxobacter*. Almost all OTUs (98.18%) were shared by *indica* and *japonica* rice, indicating minimal differences in the bacteria composition (Fig 1D, S3 Fig). About 13 core microorganisms including *Rivibacter*, *Synechococcus*, and *Bacillus*, were observed among the soil samples where rice was planted.

**Fig 2:**
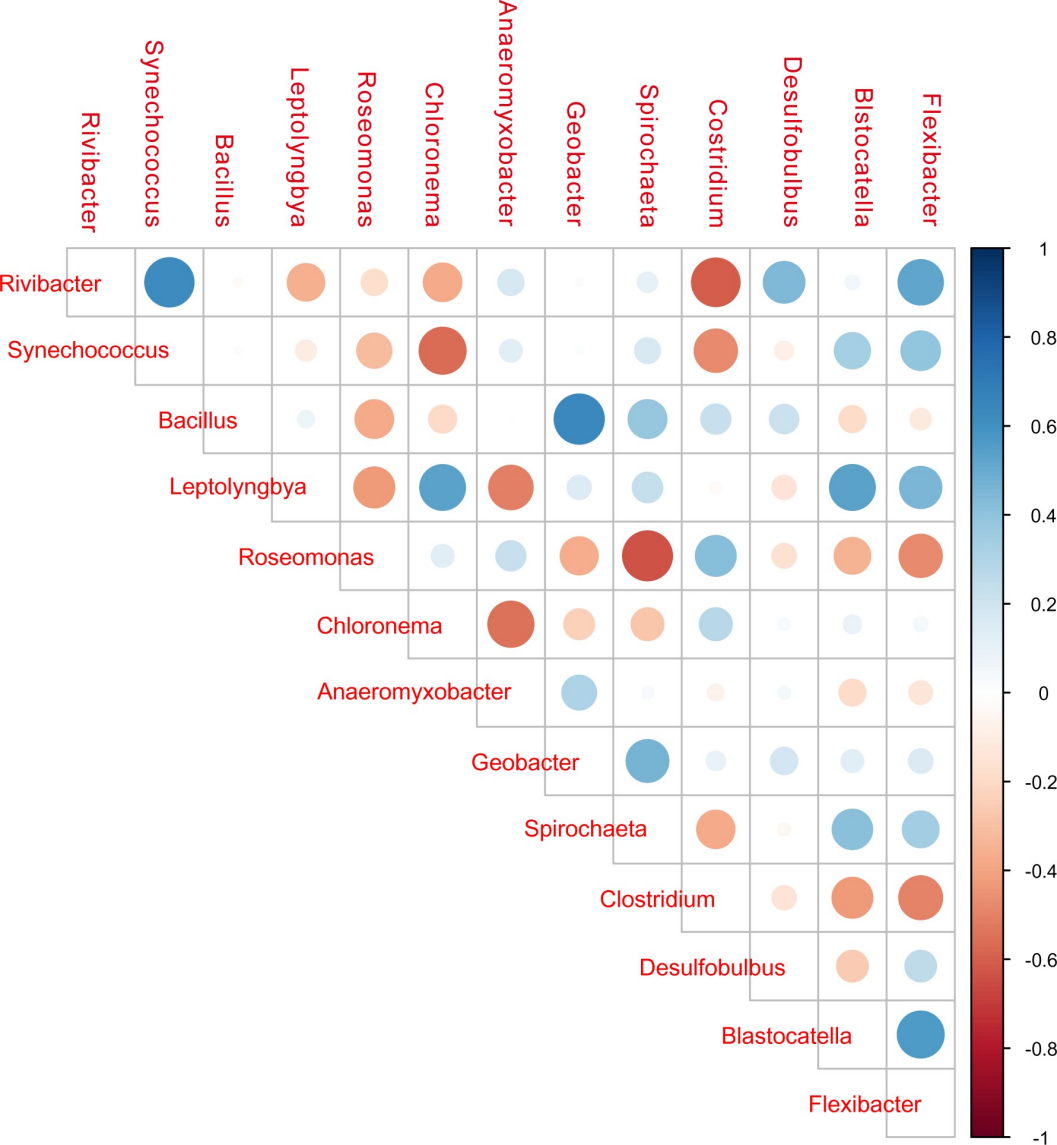
Correlations among the core bacteria. Correlation of two bacterial genera was shown in locations with crosses. Blue colors showed positive correlations, while reddish hues showed negative correlations; dots with larger diameters showed stronger correlations.

Correlations of these primary microorganisms were calculated based on Spearman rank correlation. Positive correlations were found between *Bacillus* and *Geobacter*; *Rivibacter* and *Synechococcus*; and *Leptolyngbya* with *Chloronema*, *Blastocatella* and *Flexibacter*. *Leptolyngbya* was negatively correlated with *Roseomonas* and *Anaeromyxobacter*. *Flexibacter* was positively correlated with most core bacteria (Fig 2). Positive correlations indicated growth promotion (synergistic) and negative correlations indicated inhibition (antagonistic).

### Comparison of bacterial community structure among groups

**Fig 3:**
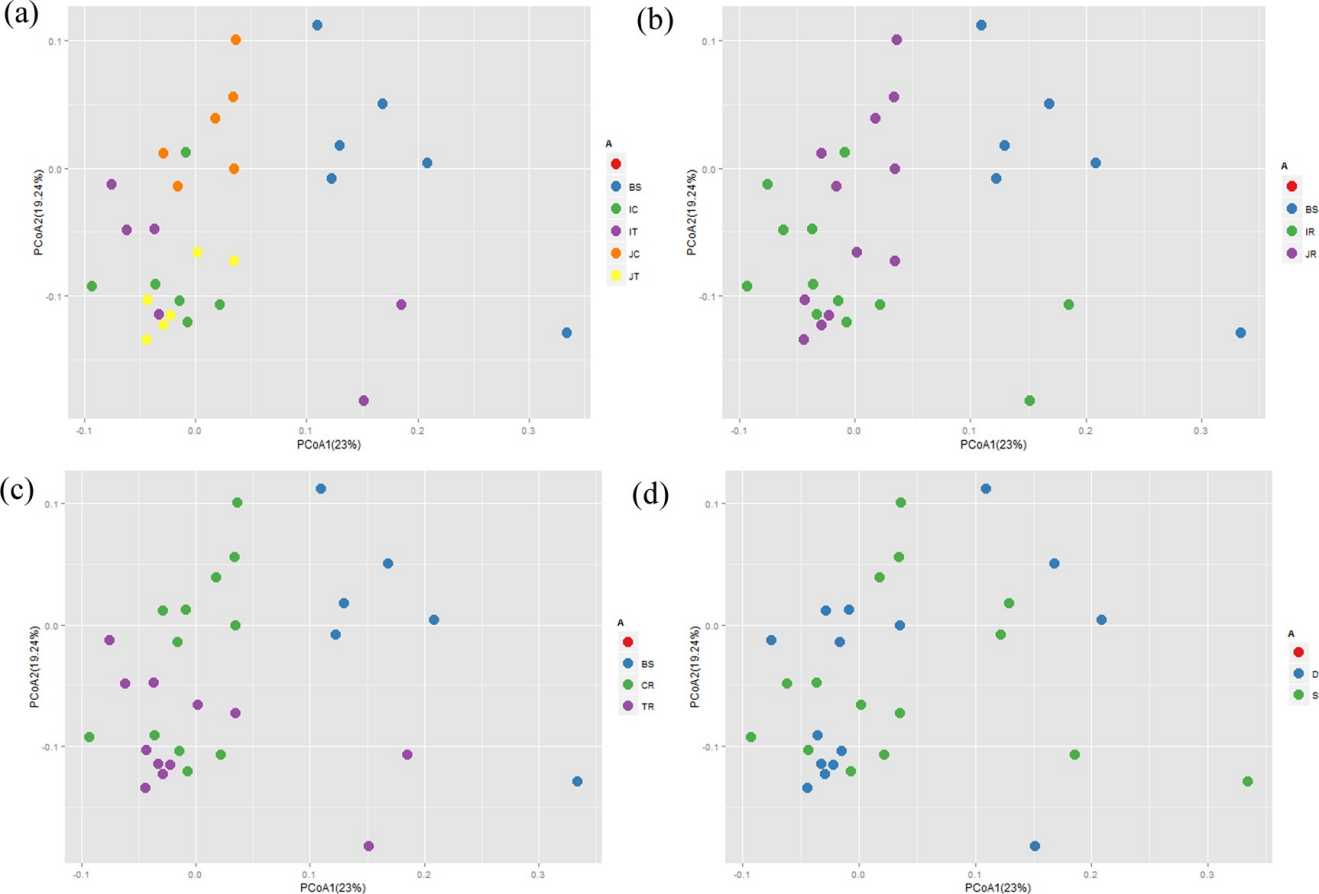
Principal coordinate analysis of different classifications based on weighted UniFrac distance. (a), principal coordinate analysis of group samples categorized by genetic modification status and rice subspecies. (b), principal coordinate analysis for different groups of rice subspecies. (c), principal coordinate analysis of the GM group samples and non-GM group samples. (d), principal coordinate analysis of topsoil samples and subsoil samples.BS, blank soil; IT, transgenic *indica* rice, IC, *indica* rice control; JT, transgenic *japonica* rice and JC for *japonica* rice control; JR, *japonica* rice, IR, *indica* rice; TR, transgenic rice, CR, conventional rice control; D, subsoil soil; S, topsoil.

Samples were divided into different groups based on cultivar, genetic modification status, and difference of soil depth. Principal coordinate analysis (PCoA) was used to identify the community structure differences among different groups. Cluster analysis and the PCoA maps were constructed based on weighted UniFrac (Fig 3) and unweighted UniFrac (S4 Fig).

When all tested samples were divided into five groups according to the cultivar and genetic modification status, the *japonica* pair were clearly distinct from each other, but *indica* pair were in a cluster (Fig 3A). While samples were separated into blank, *japonica*, and *indica* groups, the *japonica* and *indica* groups did not form two distinct clusters based on weighted UniFrac of PCoA analysis (Fig 3B). Similarly, transgenic and conventional groups were not separated (Fig 3C) and blank samples were clustered together but separated from the other groups (Fig 3A, 3B and 3C). Two groups at different soil depths were not distinct, indicating no difference between the two tested groups. Differences in bacterial community structure were only shown in *japonica* pair (*bar-*transgenic and its conventional rice soil samples); the other groups were similar.

### Dominant factors affecting the distribution of plant root microorganisms

**Fig 4:**
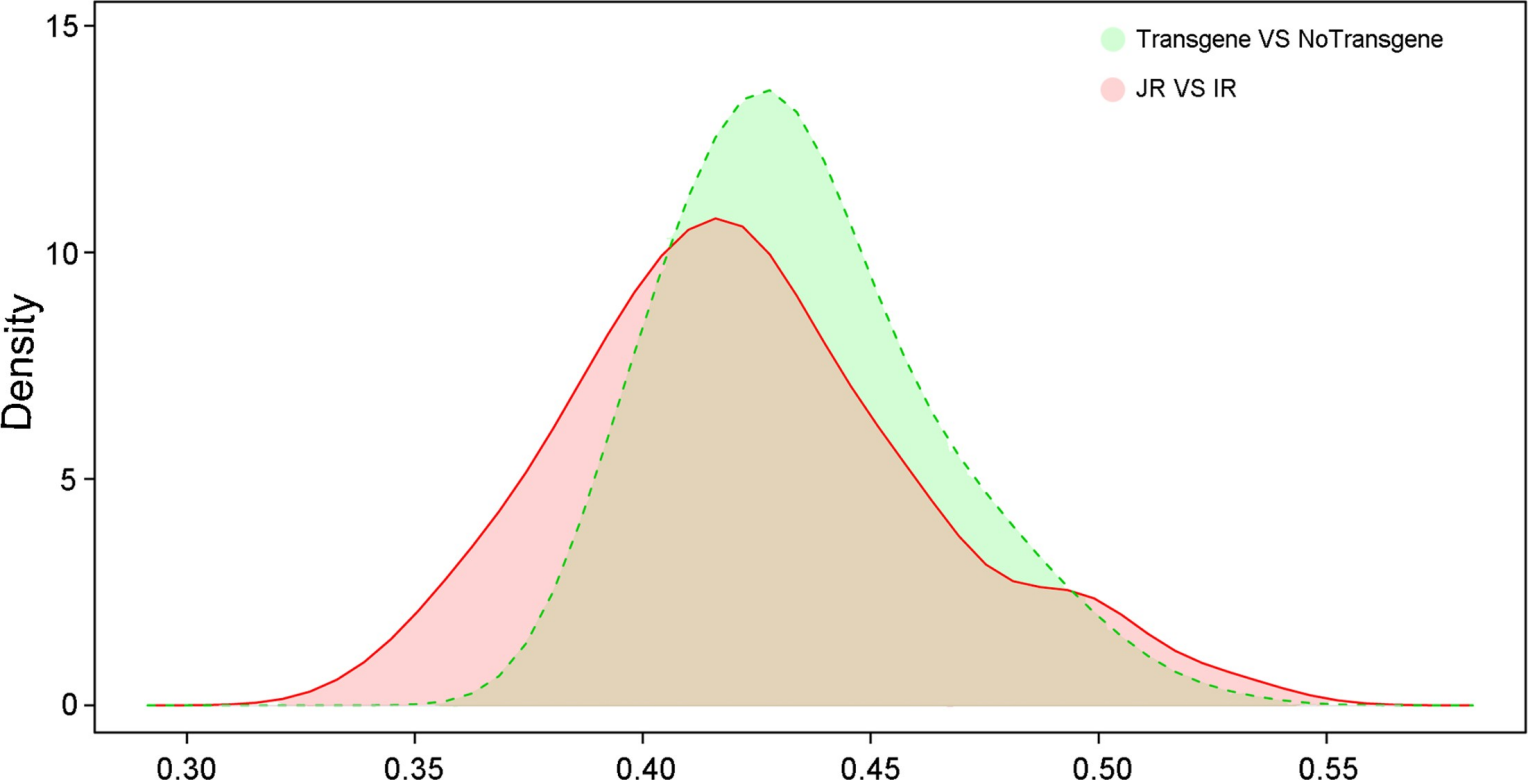
Kernel density estimation of evolutionary distance for transgenic vs non-transgenic and *japonica* vs *indica* samples. Transgene vs No Transgene, Transgenic pair and non-transgenic pair; JR vs IR, *japonica* pair and *indica* pair.

As mentioned above, rice lines and transgenes might affect the distribution of plant root microorganisms. *Indica* rice and *japonica* rice were differentiated and developed over long-term evolution in different climates and ecological environments, while transgenic rice was artificially created by inserting a gene fragment that could be stably inherited by offspring. Evolutionary distance of microorganisms was compared to find the main factor differentiating the richness of soil microorganisms. The evolutionary distance of samples from transgenic and non-transgenic groups was farther than that between subspecies rice varieties (Fig 4). This result may be attributed to foreign genes extracted from unrelated organisms.

### Analysis of root microorganisms between transgenic and non-transgenic rice

**Fig 5:**
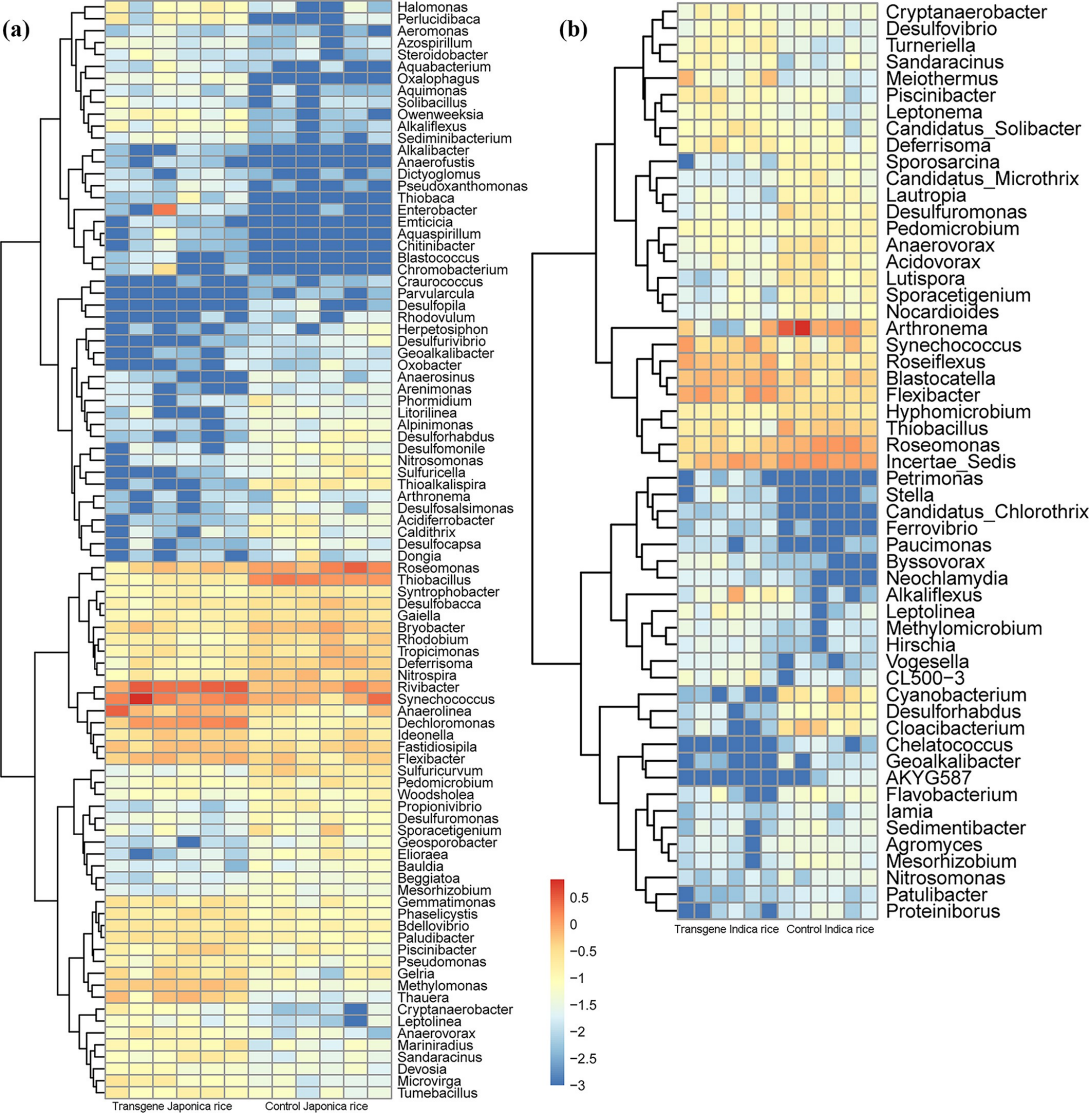
Heatmap of the classified bacterial genera of the paddy soil between transgenic and conventional rice. (a), different bacterial genera between transgenic *japonica* rice and its conventional rice. (b), different bacterial genera between transgenic *indica* rice and its conventional rice.

Since transgenes were the dominant factor affecting root microorganisms, it was important to determine where the differences originated. The *indica* rice pair and the *japonica* rice pair (GM and non-GM) were analyzed using Welch’s *t*-test (unequal variances). The result showed that there were around 92 different bacterial genera for the comparison of transgenic *japonica* rice and non-transgenic *japonica* rice, and 55 different genera for the comparison of transgenic *indica* rice and its non-transgenic *indica* rice. Differences in *japonica* rice were more obvious than for *indica* rice. *Rivibacter*, *Synechococcus*, *Anaerolinea*, *Dechloromonas*, *Thiobacillus*, and *Roseomonas* were the dominant bacterial genera in the *japonica* group, meanwhile, *Arthronema*, *Roseiflexus*, *Blastocatella*, *Flexibacter*, and *Roseomonas* were the primary bacterial genera in the *indica* pair. The *japonica* pair and the *indica* pair had 19 shared bacterial genera among the significantly different microorganisms. The relative abundance of bacterial microflora was different. Certain bacterial genera (e.g. *Synechococcus*, *Flexibacter*, *Alkaliflexus*, *Leptolinea*, *Piscinibacter*, *Sandaracinus*, and *Cryptanaerobacter*) were more abundant in transgenic rice soil samples than conventional rice soil samples. However, *Roseomonas*, *Nitrosomonas*, *Desulforhabdus*, *Arthronema*, *Desulfuromonas*, *Geoalkalibacter*, *Mesorhizobium*, *Pedomicrobium*, *Sporacetigenium*, and *Thiobacillus* were deficient in transgenic rice soil samples compared with conventional rice samples. Of the common bacterial genera, *Anaerovorax* was more abundant in transgenic *japonica* rice soil than soil from conventional rice, while it was deficient in transgenic *indica* rice soil compared to soil with conventional rice and opposite responses to *Deferrisoma* in the *indica* rice pair were observed (Fig 5).

### Soil microorganisms from transgenic root systems influenced neighboring soil

**Fig 6:**
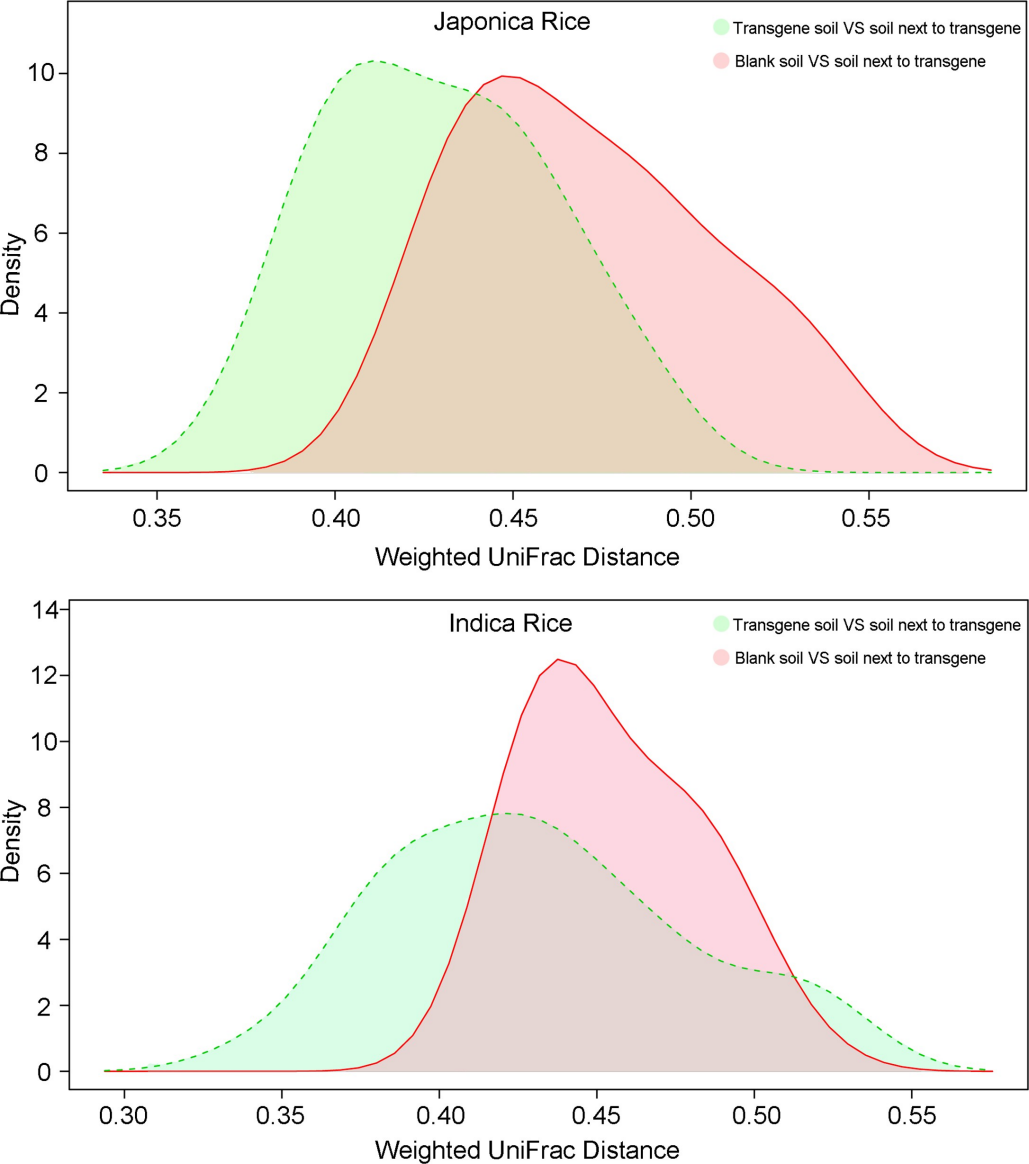
Kernel density estimation of evolutionary distance for two subspecies rice samples.

To determine the effects of transgenes on neighboring soil environments, microbial community structure in the surrounding soil was analyzed based on evolutionary distance. Both transgenic *japonica* rice and *indica* rice had a similar effect on their surrounding soil, in that microbial community structure in the surrounding soils was similar to that of the neighboring plants, but significantly different from the control blank soil (Fig 6). The results showed that the rhizosphere microorganisms of transgenic rice could affect the distribution of microorganisms in the surrounding soil.

A total of 25 bacterial communities had significantly different relative abundance at the genus level (Table 4). For example, *Leptolyngbya*, *Roseomonas*, and *Tropicimonas* from the transgenic rice rhizosphere soil were respectively about tenfold, fivefold, and three-fold less abundant than in blank soil. In contrast, *Dechloromonas*, *Fastidiosipila*, and *Cryptanaerobacter* from transgenic rice soil were more abundant than in blank soil. There were more significantly different bacterial genera between transgenic *japonica* rice soil and blank soil than that between *indica* rice soil and blank soil. These results showed clear effects of microorganisms on soil adjacent to the root system of the transgenic plant.

**Table 4:** Influence of bacterial communities from rhizosphere soil of transgenic rice on neighboring blank soil.

Genera	Relative contribution (%)		Enriched	Adjusted
	Blank	Trans		*p*-value
**Blank soil vs transgenic *japonica* rice soil**				
*Anaeromyxobacter*	0.3134	0.9868	Trans	0.0001
*Dechloromonas*	0.0271	0.6525	Trans	0.0001
*Exiguobacterium*	0.0076	0.1521	Trans	0.0001
*Fastidiosipila*	0.0247	0.324	Trans	0.0001
*Leptolyngbya*	3.6588	0.3578	Blank	0.0001
*Paludibacter*	0.1113	0.2635	Trans	0.0001
*Roseomonas*	1.1685	0.2741	Blank	0.0001
*Syntrophorhabdus*	0.096	0.3613	Trans	0.0001
*Thermomonas*	0.1429	0.0271	Blank	0.0001
*Gemmatimonas*	0.3977	0.1749	Blank	0.0002
*Lysinibacillus*	0.011	0.1116	Trans	0.0004
*Thiobacillus*	0.9636	0.1099	Blank	0.0004
*Cryptanaerobacter*	0	0.0959	Trans	0.0007
*Macellibacteroides*	0	0.0164	Trans	0.0007
*Lysobacter*	0.4553	0.1159	Blank	0.0008
*Tropicimonas*	0.4438	0.1026	Blank	0.0008
*Alkaliphilus*	0.0008	0.1038	Trans	0.0008
*Sideroxydans*	0.0011	0.0821	Trans	0.0008
*Anaerovorax*	0.0015	0.0873	Trans	0.0008
*Paenibacillus*	0.0015	0.0496	Trans	0.0008
*Gemmobacter*	0.0067	0.1343	Trans	0.0009
*Leptonema*	0.0045	0.2495	Trans	0.0009
**Blank soil vs transgenic *indica* rice soil**				
*Fastidiosipila*	0.0247	0.2742	Trans	0.0001
*Roseomonas*	1.1685	0.2233	Blank	0.0001
*Dechloromonas*	0.0271	0.1312	Trans	0.0002
*Exiguobacterium*	0.0076	0.507	Trans	0.0002
*Geobacter*	0.2237	0.5477	Trans	0.0002
*Lysobacter*	0.4553	0.4126	Blank	0.0002
*Leptolyngbya*	3.6588	0.3532	Blank	0.0004
*Phycisphaera*	0.0457	0.1771	Trans	0.0004
*Syntrophorhabdus*	0.096	0.2213	Trans	0.0004
*Cryptanaerobacter*	0	0.0628	Trans	0.0007
*Macellibacteroides*	0	0.0522	Trans	0.0007
*Tropicimonas*	0.4438	0.1213	Blank	0.0008
*Alkaliphilus*	0.0008	0.1345	Trans	0.0008
*Sideroxydans*	0.0011	0.0951	Trans	0.0008
*Anaerovorax*	0.0015	0.0587	Trans	0.0008
*Paenibacillus*	0.0015	0.0773	Trans	0.0008
*Tissierella*	0.0042	0.21	Trans	0.0009
*Leptonema*	0.0045	0.306	Trans	0.0009

### Correlation analysis between root microbes and soil physical and chemical indices

**Fig 7:**
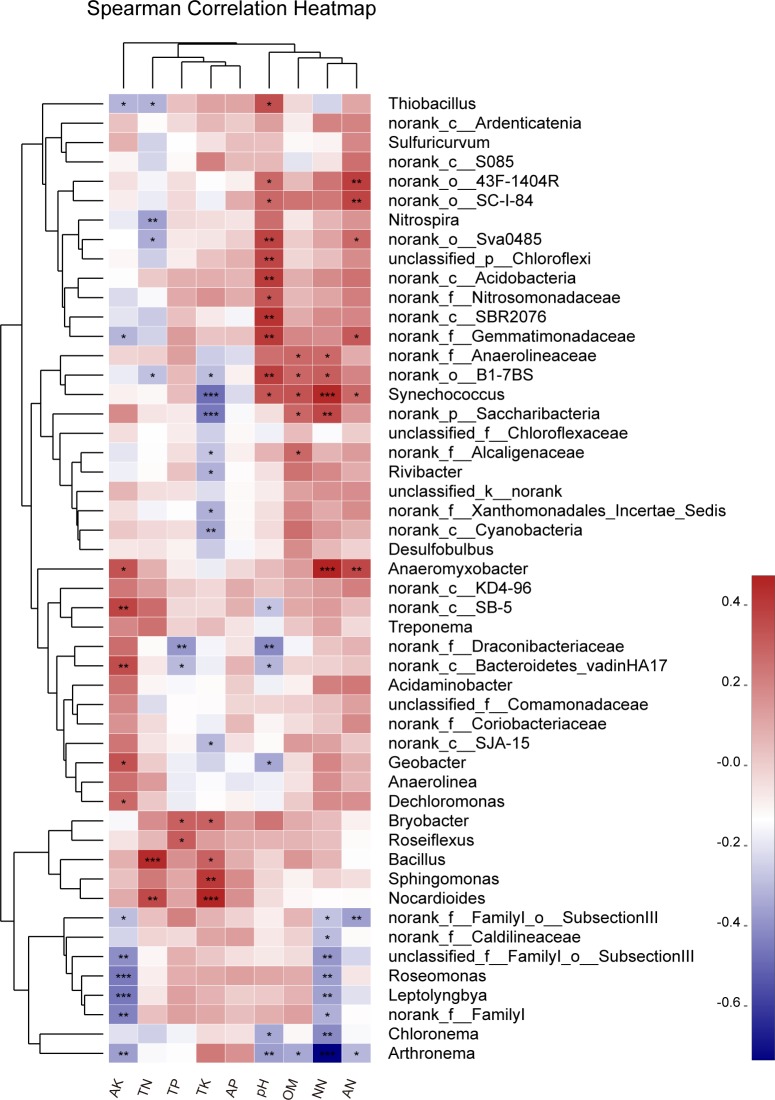
Correlations between environmental factors and the top 50 most abundant bacterial communities at genus level. AK, available potassium; AP, available phosphorus; AN, ammonium nitrogen; NN, nitrate nitrogen; OM, organic matter; TK, total potassium; TP, total phosphorus; TN, total nitrogen. The red colors represented positive correlations, while blue colors represented negative correlations. Darker colors represent stronger correlations. Significant differences were represented by: *0.01<*p* = <0.05, **0.001<*p* = <0.01, ***p<0.001.

Further analysis of correlations between environmental factors and the top 50 most abundant bacterial communities at the genus level were based on the Spearman rank correlation coefficient. Results revealed correlations between soil physical indices and bacterial communities in rice root systems (Fig 7). About 16 bacterial genera were significantly correlated with soil pH, six with negative correlations and ten with positive correlations (Table 5). Six bacterial genera were significantly correlated with soil organic matter (OM) concentration, five with positive correlations and one with negative correlations. Meanwhile, some genera were significantly correlated with the concentration of available nutrients. The concentrations of available potassium (AK), nitrate nitrogen (NN), and ammonium nitrogen (AN) were significantly correlated to 13, 13, and 8 genera, respectively. No genus was significantly correlated with available phosphorus (AP). Total potassium (TK) concentration was significantly correlated with 12 genera, but only four genera had positive correlations. The total nitrogen (TN) concentration and the total phosphorus (TP) concentration were significantly related to six and four genera, respectively. In addition, the bacterial genus *Arthronema* was significantly negatively correlated to most environmental factors, while *Bacillus* and *Anaeromyxobacter* were positively correlated to most environmental factors in this study. The correlation between other genera and environmental factors were varied. In short, environmental factors affected the bacterial community and created differences in their abundance.

**Table 5:** Correlations between environmental factors and the top 50 most abundant bacterial communities at genus level.

Environmental factor	Genus	Significant different(*0.01<*p* = <0.05,**0.001<*p* = <0.01,***p<0.001)	Negative/ positive correlation
pH	norank_f__Draconibacteriaceae	0.002**	negative
	*Geobacter*	0.01**	negative
	norank_c__Bacteroidetes_vadinHA17	0.021*	negative
	norank_c__SB-5	0.043*	negative
	*Chloronema*	0.011*	negative
	*Arthronema*	0.006**	negative
	norank_c__Acidobacteria	0.003**	positive
	norank*_*f__Nitrosomonadaceae	0.016*	positive
	norank_o__43F-1404R	0.045*	positive
	*Synechococcus*	0.016*	positive
	norank_o__B1-7BS	0.004**	positive
	norank_c__SBR2076	0.002**	positive
	norank_f__Gemmatimonadaceae	0.003**	positive
	*Thiobacillus*	0.011*	positive
	norank_o__SC-I-84	0.043*	positive
	norank_o__Sva0485	0.005**	positive
	*unclassified*_p__Chloroflexi	0.006**	positive
OM	norank_f__Anaerolineaceae	0.043*	positive
	norank_f__Alcaligenaceae	0.042*	positive
	norank_o__B1-7BS	0.04*	positive
	*Synechococcus*	0.013*	positive
	norank_p__Saccharibacteria	0.041*	positive
	*Arthronema*	0.011*	negative
TN	*Nitrospira*	0.007**	negative
	norank_o__B1-7BS	0.035*	negative
	*Thiobacillus*	0.018*	negative
	norank_o__Sva0485	0.014*	negative
	*Bacillus*	0.001**	positive
	*Nocardioides*	0.007**	positive
TP	norank*_*f__Draconibacteriaceae	0.004**	negative
	norank_c__Bacteroidetes_vadinHA17	0.025*	negative
	*Bryobacter*	0.029*	positive
	*Roseiflexus*	0.024*	positive
TK	norank*_*f__Xanthomonadales_Incertae_Sedis	0.015*	negative
	norank_f__Alcaligenaceae	0.043*	negative
	norank_c__Cyanobacteria	0.008**	negative
	norank_c__SJA-15	0.023*	negative
	*Rivibacter*	0.016*	negative
	norank_o__B1-7BS	0.03*	negative
	*Synechococcus*	0***	negative
	norank_p__Saccharibacteria	0***	negative
	*Bacillus*	0.034*	positive
	*Sphingomonas*	0.002**	positive
	*Bryobacter*	0.031*	positive
	*Nocardioides*	0***	positive
AK	*unclassified*_f__FamilyI_o__SubsectionIII	0.002**	negative
	*Leptolyngbya*	0***	negative
	*Thiobacillus*	0.02*	negative
	norank_f__Gemmatimonadaceae	0.021*	negative
	norank_f__FamilyI_o__SubsectionIII	0.03*	negative
	*Roseomonas*	0.001***	negative
	norank*_*f__FamilyI	0.001***	negative
	*Arthronema*	0.007**	negative
	*Geobacter*	0.015*	positive
	norank*_*c__Bacteroidetes_vadinHA17	0.009**	positive
	*Anaeromyxobacter*	0.015*	positive
	norank_c__SB-5	0.006**	positive
	*Dechloromonas*	0.047*	positive
NN	norank_f__Caldilineaceae	0.029*	negative
	*unclassified*_f__FamilyI_o__SubsectionIII	0.004**	negative
	*Leptolyngbya*	0.007**	negative
	norank*_*f__FamilyI_o__SubsectionIII	0.038*	negative
	*Roseomonas*	0.006**	negative
	norank_f__FamilyI	0.016*	negative
	*Chloronema*	0.002**	negative
	*Arthronema*	0***	negative
	norank_f__Anaerolineaceae	0.047*	positive
	*Anaeromyxobacter*	0***	positive
	norank_o__B1-7BS	0.025*	positive
	*Synechococcus*	0.001***	positive
	norank_p__Saccharibacteria	0.006**	positive
AN	norank_f__FamilyI_o__SubsectionIII	0.006**	negative
	*Arthronema*	0.023*	negative
	*Anaeromyxobacter*	0.006**	positive
	norank_o__43F-1404R	0.003**	positive
	*Synechococcus*	0.045*	positive
	norank_f__Gemmatimonadaceae	0.02*	positive
	norank_o__SC-I-84	0.005**	positive
	norank_o__Sva0485	0.047*	positive
AP	none	/	/

AK, available potassium; AP, available phosphorus; AN, ammonium nitrogen; NN, nitrate nitrogen; OM, organic matter; TK, total potassium; TP, total phosphorus; TN, total nitrogen.

## Discussion

Biological diversity including composition, structure, and function [40], which was rapidly lost with the development of agriculture. Taking advantage of biodiversity can benefit agricultural production [41]. As a large number of GM crops come out, the effect from cultivation of GM crops to biological diversity had raised concern. However, scientists were considered that GM crops seriously reduce biodiversity and damage the environment [42]. Whether GM crops will affect the soil microbial community, it has become an issue of public concern [43].

In response to this concern, scientists had carried out related research. Insect resistant *Bt* maize, for example, which was the most important transgenic crop did not change the microbial populations of the soil or in the activity of the microbial community [11], the rhizosphere bacterial community [15] and the soil microarthropod communities [44]. Similarly, *Bt* cotton cultivation did not affect microbial communities in the rhizosphere soil [14] and the community characteristics of rhizosphere soil nematodes [13]. And *Cry1Ac* transgenic sugarcane would not change the diversity of microbial communities or modify enzyme activities in rhizosphere soil within one crop season [45]. *Bt* rice also had no persistent or adverse effect on the microbial community composition in its rhizosphere [12, 29]. However, planting *Bt* rice had the potential to modify bacterial abundances and community structures [19], the methanogenic community composition [22] in their rhizosphere and a strong influence on active methanogens [46]. And for other functional traits of genetically modified crops, transgenic potato roots with T4 lysozyme released had no deviation in the rhizosphere communities compared to the control lines [10]. T8-ipt (isopentenyl transferase) rice cannot affect the soil bacteria biomass during growth of plants in the field [47]. Beta-carotene transgenic rice with four synthetic genes had no specific effect on rhizosphere enzyme activities and bacterial communities at different growth stages [18]. Salt tolerant *SUV3* rice sustained a healthy ecology and usual functions of the rhizospheric organisms [17]. Transgenic *Brassica napus* containing the antifungal synthetic chitinase (NiC) may not affect the enzyme activities and community structure of microbes in rhizosphere soil [48]. GM eggplants had a short-term impact on soil quality and microbial diversity and the differences disappeared post-harvest [49]. The cultivation of transgenic tobacco can affect rhizosphere/ rhizoplane microbial communities during early plant development, but the original bacterial diversity would restore after one cycle of plant cultivation [21], while transgenic tobacco plants that express and release extracellular microbial phytases from their roots, could not change the microbial community in the rhizosphere [50]. Altogether, most *Bt* and other transgenic crops had no/little effect on the soil microbial community.

Herbicide resistance was a dominant trait of GM crops, and it was widely used transgene research. In this way, it was more important to evaluate the effect of GM crops with herbicide resistance on the soil microbial community. *EPSPS*-transgenic soybean unleashed temporary effects on the taxonomic diversity of rhizosphere bacterial communities at the vegetative and seed-filling stages compared to its recipient cultivar under field conditions, and main symbiotic nitrogen-fixing bacterial genera in the roots evidently changed from the flowering stage to the seed-filling stage [51]. No effect of the bacterial community composition in the rhizosphere was detected between herbicide-resistant maize with the *pat*-gene and its non-transgenic cultivar [52]. Using a conventional culture technique and culture-independent molecular methods to assess soil microbial community in the rhizosphere soil of herbicide resistant genetically modified Chinese cabbage, the bacterial, fungal and actinomycetes population densities, DGGE banding patterns and bacterial species diversity indices were all similar to those of the non-GM Chinese cabbage soils [53] with the result of the total counts of bacteria, fungi, and actinomycetes, dominant members and PLFA (phospholipid fatty acid) profiles in the soil microbial community, Sohn. et al. believed that the herbicide-resistant *bar*-transgenic perilla had insignificant impact on the soil microbial communities when compared with those of conventional perillas [54]. However, despite these findings, there have been few studies of whether the *bar* gene or transgenic crops with herbicide resistance affected soil microorganisms. Transgenic rice that highly resistant to protoporphyrin oxidase (PPO)-inhibiting herbicides, was used to evaluate the structure of soil microbial communities, and showed no adverse effects in paddy field [9]. Similar results for cultivating transgenic rice *MSRB2-Bar-8* were reported by Sohn et al. [16]. These two researches only paid attention to the effects on transgenic *japonica* rice and their conventional rice varieties, in this study, comparison of two ecological subspecies rice (*indica* rice and *japonica* rice) and different soil layers during maturing period were both concern to affect the soil bacterial community.

Plants and soil have a variety of interactions, due to complicated environmental factors, including climate and soil characteristics. In this research, the organic matter, total phosphorus, and available phosphorus content were not significantly different among treated groups, and soil pH values were almost the same, it was agreed with the results of salt tolerant *SUV3* overexpressing transgenic rice [17] and *CaMSRB2*-expressing transgenic rice [16], except for difference in *indica* pair at the TD and SC soil layer. Total nitrogen and total potassium were in a similar way and showed difference at the TC soil layer. Soil available potassium was significant different between *bar*-*indica* rice and its conventional rice at TC and SD. Ammonium nitrogen and nitrate nitrogen were quite different between *bar*-*japonica* rice and its conventional rice at each treated groups. And the Ammonium nitrogen content of *bar*-*indica* rice soil was significantly lower than in conventional rice soil at the SD soil layer. The overall analysis of these chemical characteristics showed some significant differences between the soils of GM and non-GM rice. Moreover, soil pH was clear, significant differences between *japonica* rice and *indica* rice soils at each sampling zone. It is well known that pH plays an important role on soil microbial composition and biogeochemical function [55, 56]. For ammonium nitrogen and nitrate nitrogen, significant differences were observed between *japonica* rice and *indica* rice soils. It may be the reason that *indica* and *japonica* rice plants responded differently to NO_3_^-^, but similarly to NH_4_^+^ [57]. Some significant differences of chemical characteristics between *japonica* rice and *indica* rice soils were also observed. Nitrogen-use efficiency of *indica* varieties of rice was better than that of *japonica* varieties. NRT1.1B, a rice nitrate transporter and sensor, which is associated with the recruitment of a large proportion of *indica*-enriched bacteria, contributes to the variation in the root microbiota of *indica* and *japonica* [58].

Through the Kernel density estimation, we observed that transgenic varieties and non-transgenic varieties had larger evolutionary distances than subspecies rice varieties, suggesting that the effect from transgenes was much stronger than the effect of subspecies. This result may be attributed to foreign genes extracted from unrelated organisms. Similarly, transgenic rice containing *cry1Ab* had potential risks of modification of the methanogenic community composition in its rhizosphere [22]. Certain bacterial genera (e.g. *Synechococcus*, *Flexibacter*, *Alkaliflexus*, and *Cryptanaerobacter*) were abundant in transgenic rice but deficient in soil around conventional rice. In contrast, *Roseomonas*, *Nitrosomonas*, *Desulforhabdus*, and *Sporacetigenium* were deficient in transgenic rice soil samples compared with conventional rice samples. This phenomenon was clear with *japonica* rice. Thus, several bacterial communities strongly influence the differences, which requires further study of the impact of GM crops. The soil microenvironment was extremely complex, meaning that some small but significant differences were most likely unnoticed.

In this study, *Bacillus* was positively correlated with *Geobacter*, and species of *Bacillus* and *Geobacter* were known to reduce or sorb hexavalent chromium [59]. *Arthronema* was significantly negatively correlated to most environmental factors, while *Bacillus* and *Anaeromyxobacter* were positively correlated to most environmental factors (Fig 7). *Flexibacter* is a Gram-negative bacterium that is usually strictly aerobic and *F*. *canadensis* isolated from soil environments denitrifies NO_3_^-^ and NO_2_^-^ to gaseous forms with tolerance to oxygen [60]; *Flexibacter* was not significantly correlated with the nitrate nitrogen (NN) concentration in this study. The activity of microorganisms from the rice rhizosphere varied in different bacterial genera depending on environmental stress [61]. George et al. [50] confirmed that soil microorganisms were involved in controlling the phosphorus available to plants and that the microbial community in the rhizosphere appeared to be resistant to the impacts of inserting single genes in plants to change rhizosphere biochemistry and nutrient cycling. However, we observed that there was no significant correlation between AP and the top 50 bacterial genera. In a previous study, bacterial abundance was positively associated with soil pH [19], and pH differences were the main factor influencing soil archaeal diversity and community structure in the tropical zone and chemoautotrophic carbon dioxide fixation in drained paddy soils [62, 63]. Environmental factors clearly affected the bacterial community in this study and were the main reason for their differences in abundance. The results indicated that the differences found in the soil microbial structure between GM and non-GM rice soil, which will not be a result of the cultivation of the GM rice, but soil characteristics.

Microbial community of rice root system influences ecosystem functioning is the key to improving crop health and sustainable productivity of paddy ecosystems, and reducing methane emissions [64]. Most studies on the rhizosphere have paid more attention to the number and diversity of bacterial taxa in independent rhizospheres rather than effects from one rhizosphere to others [65]. Our study focused on rhizosphere and adjacent soil. Further research could focus on rhizosphere competition between transgenic rice and conventional rice to identify key growth stages in different areas. In this study, several bacterial communities were found to influence differences, further research is needed to determine why these differences occur.

## Conclusion

This study represented the first ecological risk assessment of the potential effects of herbicide resistance rice cultivation between two rice subspecies with different soil layers on rhizosphere microbial communities. There was no significant difference on the diversity indices between transgenic and non-transgenic rice soils, but the primary bacterial genera in *indica* rice soil were different from those in *japonica* rice. Moreover, the abundances of genera between GM and non-GM varieties in *japonica* rice were significantly different from *indica* rice, and several bacterial communities influenced these differences. *Anaerovorax* was more abundant in transgenic *japonica* rice soil than conventional soil, while it was deficient in transgenic *indica* rice soil compared to conventional rice, and opposite responses to *Deferrisoma* were in that of *indica* rice. Thus, we concluded that transgenic *indica* and *japonica* rice had different effects on soil bacteria compared with their corresponding sister conventional rice. However, these effects only occurred for a few genera but had no effect on the primary genera and soil characteristics were the main factor driving these differences. Therefore, differences in bacterial structure can be ignored when evaluating the impacts of transgenic rice on the complex soil microenvironment.

## Supporting information

S1 Table Description for acronyms. (DOCX)

S2 Table Sequence description of all samples. (DOCX)

S3 Table Alpha diversity of soil samples. (DOCX)

S1 Fig The axonometric drawing and profile map for sampling in the field. (a), axonometric drawing for soil sampling in the rice field. (b), profile map for soil sampling in the rice field. (c), profile map for sampling blank soil. **TC** was topsoil near rice basal part of rice stem while **TD** was topsoil far from rice basal part of rice stem; **SC** was subsoil near rice basal part of rice stem and **SD** was subsoil far from rice basal part of rice stem; **BTS** was blank topsoil and **BSS** was blank subsoil, neither of which have rice.
(TIF)

S2 Fig The rarefaction curve and the shannon index curve for all sequenced samples. (a), the rarefaction curve for the 54 sequenced samples. (b), the shannon index curve for the 54 sequenced samples.
(TIF)

S3 Fig Venn diagrams. (a), the shared and unique OTU among *indica*, *japonica*, and blank soil. (b), the shared and unique species among *indica*, *japonica*, and blank soil. (c), the common and unique OTU among conventional, transgenic, and blank soil. (d), the common and unique species among conventional, transgenic, and blank soil. (e), the identical and unique OTU among blank topsoil, crop topsoil, blank subsoil, and crop subsoil. (f), the identical and unique species among blank topsoil, crop topsoil, blank subsoil, and crop subsoil.
(TIF)

S4 Fig Principal coordinate analysis of different groups based on unweighted UniFrac distance. (a), principal coordinate analysis of group samples categorized by genetic modification status and rice subspecies. (b), principal coordinate analysis for different groups of rice subspecies. (c), principal coordinate analysis of the GM group samples and non-GM group samples. (d), principal coordinate analysis of topsoil samples and subsoil samples.
(TIF)
