# Supplementary material for: High-throughput sequencing analysis of microbial community diversity in response to indica and japonica bar-transgenic rice paddy soils
Source: PLoS One. 2019 Sep 9;14(9):e0222191. doi: 10.1371/journal.pone.0222191 (PMC6733487; doi:10.1371/journal.pone.0222191)
Supplement: S1 Table — (DOCX) [file pone.0222191.s001.docx]

Table S1 Description for acronyms

| **Abbreviation** | **Full name of abbreviated noun** | **Attribution category** |
| --- | --- | --- |
| GM | Genetically modified | Transgene |
| *Bt* | Bacillus thuringiensis | gene |
| *bar* | Bialaphos resistance | gene |
| TC | Topsoil of concentrated roots | Sampling soil layer |
| TD | Topsoil of dispersed roots | Sampling soil layer |
| SC | Subsoil of concentrated roots | Sampling soil layer |
| SD | Subsoil of dispersed roots | Sampling soil layer |
| OM | Organic matter | Soil chemical properties |
| TN | Total nitrogen | Soil chemical properties |
| TP | Total phosphorus | Soil chemical properties |
| TK | Total potassium | Soil chemical properties |
| AN | Ammonium nitrogen | Soil chemical properties |
| NN | Nitrate nitrogen | Soil chemical properties |
| AP | Available phosphorus | Soil chemical properties |
| AK | Available potassium | Soil chemical properties |
