# Supplementary material for: High-throughput sequencing analysis of microbial community diversity in response to indica and japonica bar-transgenic rice paddy soils
Source: PLoS One. 2019 Sep 9;14(9):e0222191. doi: 10.1371/journal.pone.0222191 (PMC6733487; doi:10.1371/journal.pone.0222191)
Supplement: S2 Table — (DOCX) [file pone.0222191.s002.docx]

Table S2 Sample sequence description

| **Samples** | **Raw reads** | **Valid reads** | **Bases(bp)** | **Length(bp)** | **OTU** |
| --- | --- | --- | --- | --- | --- |
| **BTS** | 39229 ± 5541 | 31381 ± 7805 | 13626659 ± 3322923 | 434.6 ± 2.4 | 2682.0 ± 278.1 |
| **BSS** | 39870 ± 7601 | 31334 ± 6745 | 13662804 ± 2934376 | 436.1 ± 0.4 | 2804.7 ± 278.0 |
| **TJTC** | 29456 ± 5682 | 23035 ± 4031 | 10066305 ± 1732704 | 437.1 ± 1.2 | 2563.3 ± 38.6 |
| **TJTD** | 40464 ± 8635 | 32733 ± 8017 | 14356814 ± 3527615 | 438.6 ± 0.7 | 2600.3 ± 244.8 |
| **TJSC** | 36277 ± 6664 | 29051 ± 4255 | 12719011 ± 1849446 | 437.9 ± 0.5 | 2639.3 ± 458.3 |
| **TJSD** | 35293 ± 7734 | 28880 ± 6002 | 12646522 ± 2646734 | 437.8 ± 0.7 | 2892.3 ± 193.4 |
| **CJTC** | 39192 ± 11334 | 31856 ± 9115 | 13950291 ± 3976474 | 438.0 ± 0.5 | 3035.7 ± 255.2 |
| **CJTD** | 41142 ± 5384 | 33172 ± 5650 | 14521927 ± 2518046 | 437.6 ± 1.4 | 2687.0 ± 337.8 |
| **CJSC** | 38892 ± 2859 | 29542 ± 4751 | 12937833 ± 2121575 | 437.8 ± 1.5 | 2688.0 ± 337.2 |
| **CJSD** | 39063 ± 6045 | 32111 ± 6299 | 14082870 ± 2760881 | 438.6 ± 0.8 | 2923.7 ± 115.8 |
| **TITC** | 37765 ± 11982 | 31795 ± 9616 | 13888460 ± 4175336 | 437.0 ± 2.5 | 2916.3 ± 288.1 |
| **TITD** | 29029 ± 4494 | 22115 ± 1977 | 9640870 ± 883590 | 435.9 ± 2.0 | 2384.0 ± 380.0 |
| **TISC** | 42156 ± 2577 | 33919 ± 7032 | 14849174 ± 3077768 | 437.8 ± 0.6 | 2940.7 ± 134.3 |
| **TISD** | 43974 ± 1369 | 34676 ± 2739 | 15164129 ± 1185962 | 437.3 ± 0.7 | 2964.7 ± 345.3 |
| **CITC** | 35837 ± 7328 | 26252 ± 3225 | 11438028 ± 1418020 | 435.7 ± 0.6 | 2104.3 ± 303.5 |
| **CITD** | 38989 ± 7305 | 28183 ± 6561 | 12336225 ± 2868814 | 437.7 ± 0.1 | 2638.7 ± 378.2 |
| **CISC** | 39471 ± 2374 | 28696 ± 1630 | 12580617 ± 745064 | 438.4 ± 1.1 | 2350.3 ± 326.2 |
| **CISD** | 41314 ± 4402 | 34737 ± 5307 | 15163554 ± 2325480 | 436.5 ± 0.3 | 3131.3 ± 147.9 |

BTS, topsoil of blank soil without planting rice; BSS, subsoil of blank soil without planting rice; TJTC, topsoil of concentrated roots from bar-transgenic japonica rice B2; TJTD, topsoil of dispersed roots from Bar-transgenic japonica rice B2; TJSC, subsoil of concentrated roots from bar-transgenic japonica rice B2; TJSD, subsoil of dispersed roots from bar-transgenic japonica rice B2; CJTC, topsoil of concentrated roots from conventional japonica rice Xiushui63; CJTD, topsoil of dispersed roots from conventional japonica rice Xiushui63; CJSC, subsoil of concentrated roots from conventional japonica rice Xiushui63; CJSD, subsoil of dispersed roots from conventional japonica rice Xiushui63; TITC, topsoil of concentrated roots from bar-transgenic indica rice B68-1; TITD, topsoil of dispersed roots from bar-transgenic indica rice B68-1; TISC, subsoil of concentrated roots from bar-transgenic indica rice B68-1;TISD, subsoil of dispersed roots from bar-transgenic indica rice B68-1; CITC, topsoil of concentrated roots from conventional indica rice D68; CITD, topsoil of dispersed roots from conventional indica rice D68; CISC, subsoil of concentrated roots from conventional indica rice D68; CISD, subsoil of dispersed roots from conventional indica rice D68. Data was shown by the average of samples (n=3) and their standard deviation.
