# Supplementary material for: High-throughput sequencing analysis of microbial community diversity in response to indica and japonica bar-transgenic rice paddy soils
Source: PLoS One. 2019 Sep 9;14(9):e0222191. doi: 10.1371/journal.pone.0222191 (PMC6733487; doi:10.1371/journal.pone.0222191)
Supplement: S3 Table — (DOCX) [file pone.0222191.s003.docx]

Table S3 Diversity indices of soil samples

| **Samples** | **Sobs** | **Ace** | **Chao** | **Coverage** | **Shannon** | **Simpson** |
| --- | --- | --- | --- | --- | --- | --- |
| **BTS** | 2684 ± 276 | 3759 ± 231 | 3697 ± 248 | 0.95 ± 0.015 | 6.60 ± 0.66 | 0.01 ± 0.008 |
| **BSS** | 2802 ± 278 | 3707 ± 369 | 3674 ± 372 | 0.95 ± 0.007 | 6.91 ± 0.11 | 0.00 ± 0.001 |
| **TJTC** | 2563 ± 35 | 3658 ± 23 | 3637 ± 101 | 0.94 ± 0.011 | 6.70 ± 0.05 | 0.00 ± 0.001 |
| **TJTD** | 2594 ± 247 | 3494 ± 245 | 3481 ± 259 | 0.96 ± 0.008 | 6.41 ± 0.05 | 0.01 ± 0.000 |
| **TJSC** | 2637 ± 461 | 3545 ± 514 | 3566 ± 539 | 0.95 ± 0.009 | 6.88 ± 0.31 | 0.00 ± 0.002 |
| **TJSD** | 2891 ± 195 | 3841 ± 180 | 3795 ± 211 | 0.95 ± 0.007 | 7.02 ± 0.06 | 0.00 ± 0.000 |
| **CJTC** | 3033 ± 264 | 4021 ± 269 | 3960 ± 231 | 0.95 ± 0.009 | 7.08 ± 0.04 | 0.00 ± 0.000 |
| **CJTD** | 2677 ± 337 | 3642 ± 259 | 3624 ± 241 | 0.96 ± 0.008 | 6.63 ± 0.26 | 0.00 ± 0.002 |
| **CJSC** | 2685 ± 345 | 3642 ± 345 | 36260 ± 373 | 0.95 ± 0.003 | 6.71 ± 0.49 | 0.01 ± 0.015 |
| **CJSD** | 2918 ± 107 | 3904 ± 92 | 3926 ± 67 | 0.95 ± 0.014 | 6.85 ± 0.36 | 0.01 ± 0.006 |
| **TITC** | 2904 ± 289 | 3914 ± 250 | 3860 ± 251 | 0.95 ± 0.009 | 6.80 ± 0.35 | 0.00 ± 0.004 |
| **TITD** | 2381 ± 381 | 3691 ± 85 | 3493 ± 423 | 0.94 ± 0.004 | 6.49 ± 0.43 | 0.01 ± 0.006 |
| **TISC** | 2930 ± 139 | 3864 ± 55 | 3868 ± 48 | 0.95 ± 0.023 | 6.99 ± 0.15 | 0.00 ± 0.001 |
| **TISD** | 2957 ± 350 | 3931 ± 374 | 3956 ± 336 | 0.95 ± 0.006 | 6.97 ± 0.18 | 0.00 ± 0.001 |
| **CITC** | 2101 ± 303 | 3053 ± 457 | 3032 ± 458 | 0.95 ± 0.008 | 6.31 ± 0.24 | 0.01 ± 0.001 |
| **CITD** | 2634 ± 379 | 3727 ± 303 | 3665 ± 316 | 0.95 ± 0.012 | 6.61 ± 0.37 | 0.01 ± 0.003 |
| **CISC** | 2353 ± 317 | 3409 ± 384 | 3385 ± 364 | 0.95 ± 0.007 | 5.87 ± 0.95 | 0.05 ± 0.058 |
| **CISD** | 3122 ± 156 | 4131 ± 147 | 4096 ± 134 | 0.96 ± 0.010 | 6.99 ± 0.18 | 0.00 ± 0.001 |

Sobs, the observed richness; ace, the ACE estimator; chao, the Chao1 estimator; Coverage, refers to the Coverage of each sample library; Shannon, the Shannon diversity index; simpson, the Simpson diversity index. BTS, topsoil of blank soil without planting rice; BSS, subsoil of blank soil without planting rice; TJTC, topsoil of concentrated roots from bar-transgenic japonica rice B2; TJTD, topsoil of dispersed roots from Bar-transgenic japonica rice B2; TJSC, subsoil of concentrated roots from bar-transgenic japonica rice B2; TJSD, subsoil of dispersed roots from bar-transgenic japonica rice B2; CJTC, topsoil of concentrated roots from conventional japonica rice Xiushui63; CJTD, topsoil of dispersed roots from conventional japonica rice Xiushui63; CJSC, subsoil of concentrated roots from conventional japonica rice Xiushui63; CJSD, subsoil of dispersed roots from conventional japonica rice Xiushui63; TITC, topsoil of concentrated roots from bar-transgenic indica rice B68-1; TITD, topsoil of dispersed roots from bar-transgenic indica rice B68-1; TISC, subsoil of concentrated roots from bar-transgenic indica rice B68-1;TISD, subsoil of dispersed roots from bar-transgenic indica rice B68-1; CITC, topsoil of concentrated roots from conventional indica rice D68; CITD, topsoil of dispersed roots from conventional indica rice D68; CISC, subsoil of concentrated roots from conventional indica rice D68; CISD, subsoil of dispersed roots from conventional indica rice D68. Data was shown by the average of samples (n=3) and their standard deviation.
