# Supplementary figures and images for: High-throughput sequencing analysis of microbial community diversity in response to indica and japonica bar-transgenic rice paddy soils
Source: PLoS One. 2019 Sep 9;14(9):e0222191. doi: 10.1371/journal.pone.0222191 (PMC6733487; doi:10.1371/journal.pone.0222191)

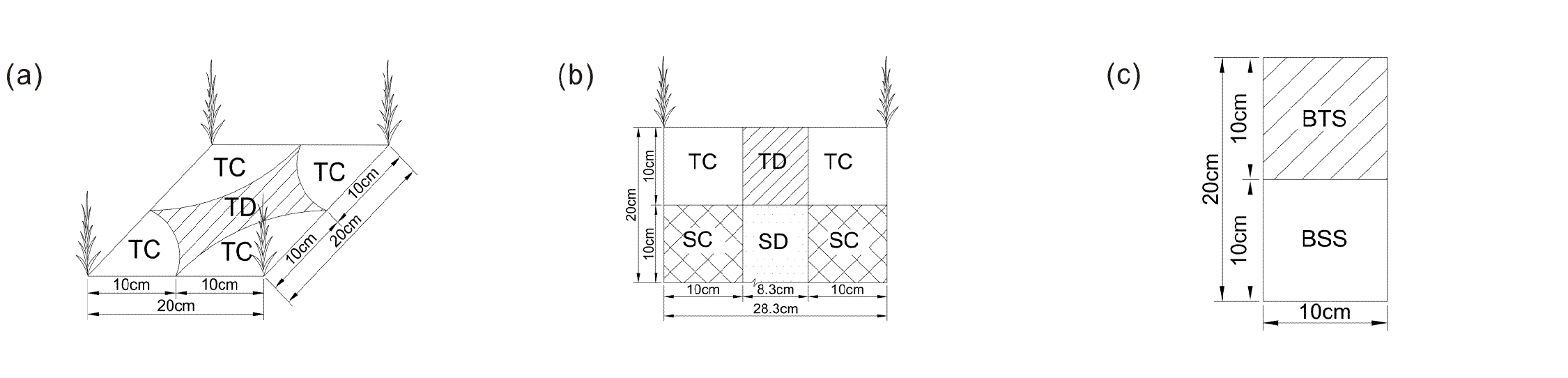

Supplement: S1 Fig — (a), axonometric drawing for soil sampling in the rice field. (b), profile map for soil sampling in the rice field. (c), profile map for sampling blank soil. TC was topsoil near rice basal part of rice stem while TD was topsoil far from rice basal part of rice stem; SC was subsoil near rice basal part of rice stem and SD was subsoil far from rice basal part of rice stem; BTS was blank topsoil and BSS was blank subsoil, neither of which have rice. (TIF) [file pone.0222191.s004.tif]

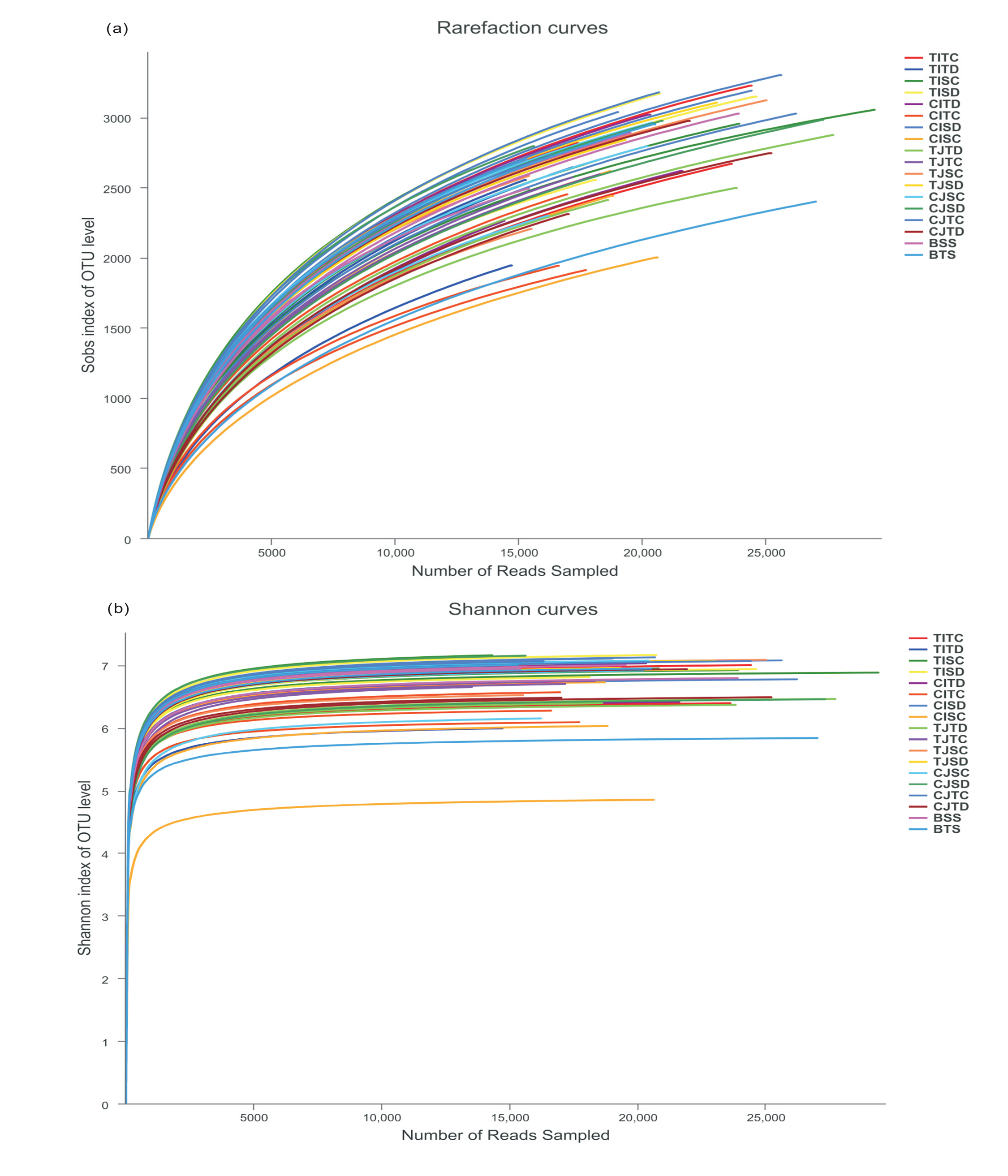

Supplement: S2 Fig — (a), the rarefaction curve for the 54 sequenced samples. (b), the shannon index curve for the 54 sequenced samples. (TIF) [file pone.0222191.s005.tif]

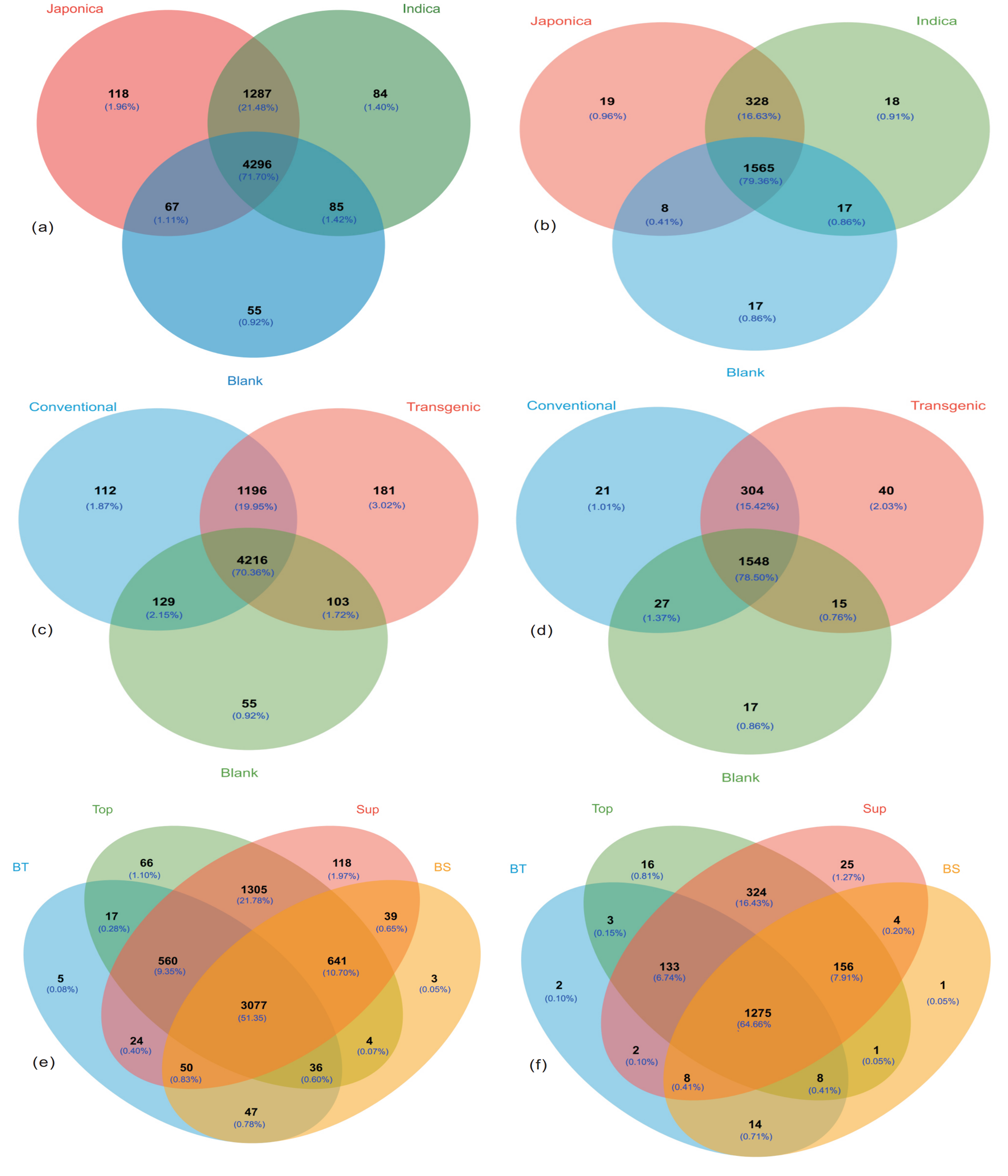

Supplement: S3 Fig — (a), the shared and unique OTU among indica, japonica, and blank soil. (b), the shared and unique species among indica, japonica, and blank soil. (c), the common and unique OTU among conventional, transgenic, and blank soil. (d), the common and unique species among conventional, transgenic, and blank soil. (e), the identical and unique OTU among blank topsoil, crop topsoil, blank subsoil, and crop subsoil. (f), the identical and unique species among blank topsoil, crop topsoil, blank subsoil, and crop subsoil. (TIF) [file pone.0222191.s006.tif]

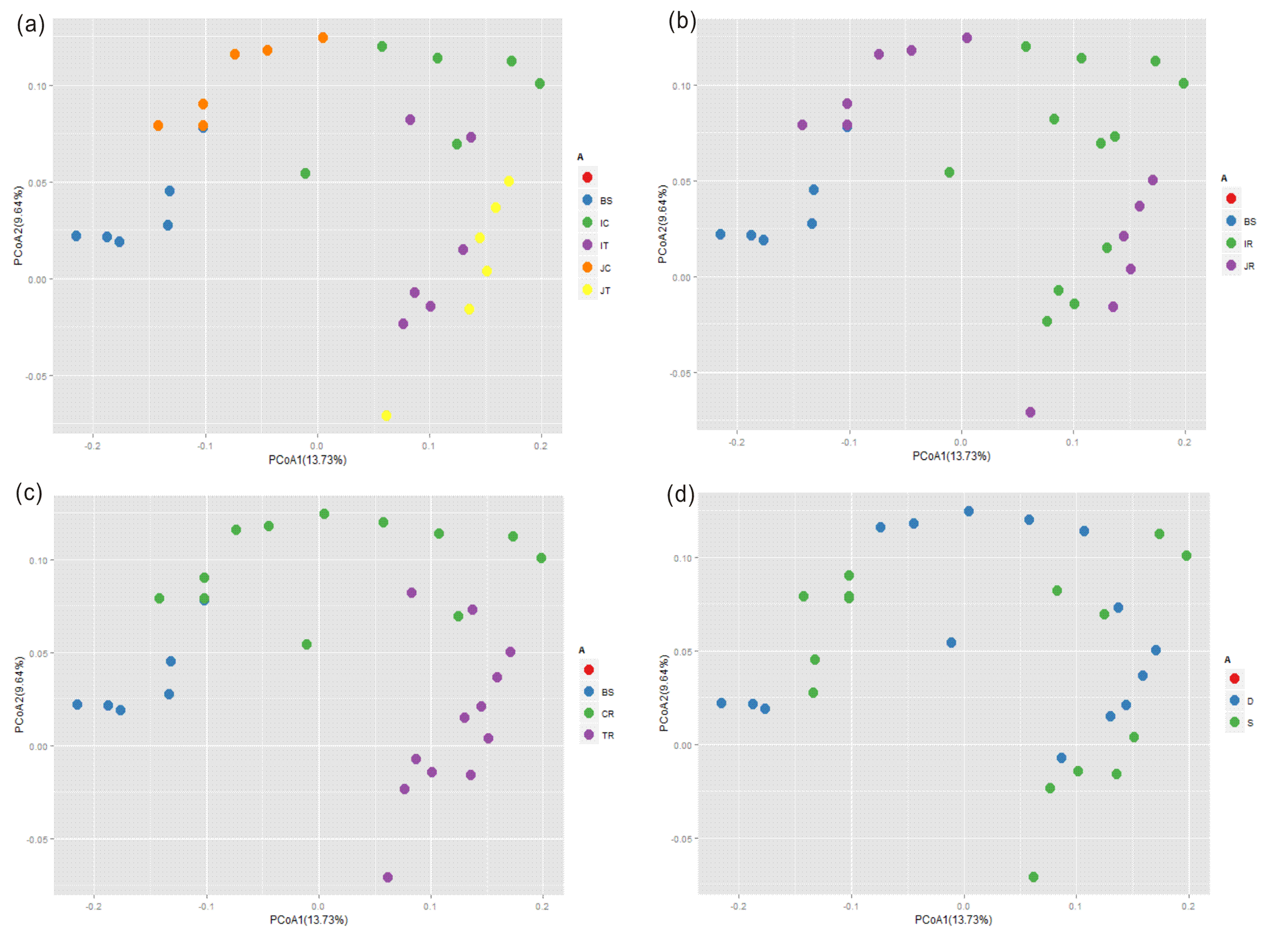

Supplement: S4 Fig — (a), principal coordinate analysis of group samples categorized by genetic modification status and rice subspecies. (b), principal coordinate analysis for different groups of rice subspecies. (c), principal coordinate analysis of the GM group samples and non-GM group samples. (d), principal coordinate analysis of topsoil samples and subsoil samples. (TIF) [file pone.0222191.s007.tif]
